# Metagenomic analysis of the gut microbiota in piglets either challenged or not with enterotoxigenic *Escherichia coli* reveals beneficial effects of probiotics on microbiome composition, resistome, digestive function and oxidative stress responses

**DOI:** 10.1371/journal.pone.0269959

**Published:** 2022-06-24

**Authors:** Prasert Apiwatsiri, Pawiya Pupa, Wandee Sirichokchatchawan, Vorthon Sawaswong, Pattaraporn Nimsamer, Sunchai Payungporn, David J. Hampson, Nuvee Prapasarakul

**Affiliations:** 1 Department of Veterinary Microbiology, Faculty of Veterinary Science, Chulalongkorn University, Bangkok, Thailand; 2 College of Public Health Sciences, Chulalongkorn University, Bangkok, Thailand; 3 Program in Bioinformatics and Computational Biology, Graduate School, Chulalongkorn University, Bangkok, Thailand; 4 Research Unit of Systems Microbiology, Department of Biochemistry, Faculty of Medicine, Chulalongkorn University, Bangkok, Thailand; 5 School of Veterinary Medicine, Murdoch University, Perth, Western Australia, Australia; 6 Center of Excellence in Diagnosis and Monitoring of Animal Pathogens, Chulalongkorn University, Bangkok, Thailand; University of British Columbia, CANADA

## Abstract

This study used metagenomic analysis to investigate the gut microbiota and resistome in piglets that were or were not challenged with enterotoxigenic *Escherichia coli* (ETEC) and had or had not received dietary supplementation with microencapsulated probiotics. The 72 piglets belonged to six groups that were either non-ETEC challenged (groups 1–3) or ETEC challenged (receiving 5ml of 10^9^ CFU/ml pathogenic ETEC strain L3.2 one week following weaning at three weeks of age: groups 4–6). On five occasions at 2, 5, 8, 11, and 14 days of piglet age, groups 2 and 5 were supplemented with 10^9^ CFU/ml of multi-strain probiotics (*Lactiplantibacillus plantarum* strains 22F and 25F, and *Pediococcus acidilactici* 72N) while group 4 received 10^9^ CFU/ml of *P*. *acidilactici* 72N. Group 3 received 300mg/kg chlortetracycline in the weaner diet to mimic commercial conditions. Rectal faecal samples were obtained for metagenomic and resistome analysis at 2 days of age, and at 12 hours and 14 days after the timing of post-weaning challenge with ETEC. The piglets were all euthanized at 42 days of age. The piglets in groups 2 and 5 were enriched with several desirable microbial families, including *Lactobacillaceae*, *Lachnospiraceae* and *Ruminococcaceae*, while piglets in group 3 had increases in members of the *Bacteroidaceae* family and exhibited an increase in *tetW* and *tetQ* genes. Group 5 had less copper and multi-biocide resistance. Mobile genetic elements IncQ1 and IncX4 were the most prevalent replicons in antibiotic-fed piglets. Only groups 6 and 3 had the integrase gene (*intl*) class 2 and 3 detected, respectively. The insertion sequence (IS) 1380 was prevalent in group 3. IS3 and IS30, which are connected to dietary intake, were overrepresented in group 5. Furthermore, only group 5 showed genes associated with detoxification, with enrichment of genes associated with oxidative stress, glucose metabolism, and amino acid metabolism compared to the other groups. Overall, metagenomic analysis showed that employing a multi-strain probiotic could transform the gut microbiota, reduce the resistome, and boost genes associated with food metabolism.

## Introduction

The gut microbiota of the pig plays a critical role in maintaining health and productivity through supporting optimal nutritional, physiological and immunological functions [1, 2]. Piglets in the weaning transition period are exposed to a variety of stressful factors that may disrupt their newly acquired gut microbiome, resulting in poor growth and health [2]. Infection with enterotoxigenic and verotoxigenic *Escherichia coli* (ETEC and VTEC) are known to cause post-weaning diarrhoea, which results in increased morbidity and mortality, decreased average daily gain (ADG), and the need for increased administration of antibiotics, which all contribute to financial losses for the pig sector [3, 4]. In response, feed additives such as antibiotics, prebiotics, and probiotics have been used to manipulate the piglet gut micro-ecosystem in order to boost growth, improve health status, and prevent diarrhoea after weaning [5].

Antibiotics have been utilized worldwide in the swine industry for many years in order to increase pig productivity while lowering morbidity and mortality [5, 6]. However, administration of in-feed antibiotics impacts both pathogenic and commensal microbes in the gut, leading to decreased alpha-diversity and causing a microbial shift in the animal gut [7]. For example, oxytetracycline treatment may diminish bacterial diversity and richness in the gut microbiota of piglets, moreover subsequent removal of oxytetracycline for 2 weeks does not completely restore bacterial diversity [8]. Several studies have found that pigs exposed to in-feed antibiotics are more likely to develop infections from members of the *Enterobacteriaceae*, *Spirochaetae*, and *Campylobacteraceae* families [6–8].

Antibiotic-treatment of piglets also can increase the diversity and abundance of antibiotic-resistant genes (ARGs) and mobile genetic elements (MGEs) in the porcine gut: these include genes conferring resistance to aminoglycosides, beta-lactams, chloramphenicol, macrolide-lincosamide-streptogramin B (MLS_B_), sulfonamides, tetracycline, and vancomycin, as well as class 1 integrons and transposons [9]. Antibiotic usage has negative consequences that may affect public health, and, as a result many countries including Thailand have banned the use of antibiotics in livestock agriculture [6]. Consequently, the use of non-antibiotic alternatives for stimulating growth and altering the gut microbiome has received considerable attention in the livestock industries [10].

Probiotics are live microorganisms that are a non-antibiotic option for maintaining gut health, and they have been thoroughly researched over the years [7]. Probiotic supplementation has been shown to have various benefits for humans and animals, including altering the gut microbiota, enhancing food utilization, strengthening gut immunity, and reducing enteric disease [5, 11, 12]. The intestinal microbiota of pigs that were supplemented with *Lactiplantibacillus plantarum* PFM105 was found to be enriched by desirable bacterial families such as *Prevotellaceae* and *Bifidobacteriaceae*, which improve nutrient absorption and have anti-inflammatory activity [7]. Pigs supplemented with 2.5×10^7^ CFU/ml of *Lactiplantibacillus plantarum* JDFM LP11 showed significantly increased gut microbial richness and diversity, and an increased *Ruminococcaceae* relative abundance of up to 25% compared to a control group [13]. The effects of probiotics on decreasing the human gut resistome have been studied [14]. For example, infants who received *Bifidobacterium longum* subsp. *infantis* EVC001 had a 90% reduction in ARG abundance when compared to a control group [14]. Unfortunately, to date there have been relatively few comparable studies on the effect of probiotics on modulating the pig gut resistome [15]. Importantly, studies on the pig resistome may provide better insight into antimicrobial resistance (AMR) issues that impact on AMR transmission from pigs to pork consumers.

In our previous studies, several probiotic strains, including *Lactiplantibacillus plantarum* strains 22F and 25F (L22F and L25F) and *Pediococcus acidilactici* strain 72N (P72N), showed excellent safety features, including lack of antimicrobial-resistance genes based on the European Food Safety Authority (EFSA) criteria [16]. Furthermore, they demonstrated promising antibacterial, antiviral, anticonjugation, and antibiofilm action *in vitro* [17–19]. In addition, we previously created a method for preserving our probiotic strains in the form of double-coated microencapsulation for use in pig farms. In an *in vivo* investigation, these probiotic strains used at a final concentration at 10^9^ CFU/ml improved intestinal health and growth development in pigs during the rearing cycle [20, 21]. The purpose of the current study was to undertake whole-metagenome shotgun sequencing on faecal samples to investigate how feeding microencapsulated single-strain and multi-strain probiotics to neonatal pigs influenced their gut microbiota and modulated carriage of ARGs. The study also examined changes in the microbiota that were associated with feeding chlortetracycline or that resulted from ETEC challenge after weaning.

## Materials and methods

### Animals and housing

The experiments performed in this study were approved by the Institutional Animal Care and Use Committee of the Thai Food Research Center, Thai Foods Group (TFG) Public Company Limited (PLC.) under protocol no. 6112–01, and the Feed Research and Innovation Centre, Charoen Pokphand Foods (CPF) Public Company Limited (PLC.) under protocol no. FRIC-ACUP-1707013. All animal usage and procedures were performed in compliance with the International Guiding Principles for Biomedical Research Involving Animals. The euthanasia procedures were performed following the guidelines for the euthanasia of animals, in compliance with the American Veterinary Medical Association (AVMA). The piglets were rendered unconscious by administering intravenous sodium pentobarbital anaesthesia followed by potassium chloride to induce cardiac arrest and death. The use of all bacterial strains, including lactic acid bacteria (LAB) and ETEC, was approved by the Institutional Biosafety Committee, Chulalongkorn University under Biosafety Use Protocol numbers IBC1831044 and IBC1831045, respectively.

A total of 72 two-day-old healthy neonatal piglets (Large White × Landrace × Duroc) were recruited into the study. The production and health data for 60 of the pigs has been published elsewhere [22]. In the current study an additional 12 piglets were included as a positive control group that were administered with chlortetracycline, with these being reared and handled in an identical fashion to the previously described piglets. The 72 piglets were randomly allocated into six experimental groups with male and female replicate pens per group (6 pigs per pen) at the CPF Feed Research and Innovation Centre. At 21 days of age, piglets in all experimental groups were weaned and transferred to the TFG Research Center. Each experimental group was raised in separate rooms with controlled humidity under an evaporative cooling system at 80%. The environment within the building was temperature-controlled at 32 ± 2°C and 27 ± 1°C for neonatal and weaned piglets, respectively. All piglets were allowed to independently suck the milk from their sows in the neonatal period. They were allowed *ad libitum* access to a basal diet and water in the weaning period. The ingredient composition and nutrient concentration of the weaner diet is presented in the supplementary data (**S1 Table**).

### Experimental design and sample collection

Information about the treatments received by the six experimental groups is summarized in **Table 1** and **S1 Fig.** The three groups supplemented with probiotics received these on five occasions, when the piglets were 2, 5, 8, 11, and 14 days of age, followed our previous study [20].

**Table 1:** Summary of the experimental groups.

No.	Experimental group	Probiotic supplementation			ETEC infection	Antibiotic administration
		*P*. *acidilactici* 72N (P72N)	*L*. *plantarum* 22F (L22F)	*L*. *plantarum* 25F (L25F)		
**Non-ETEC infection**						
1	Negative control	-	-	-	-	-
2	Probiotic control	+	+	+	-	-
3	Antibiotic	-	-	-	-	+
**ETEC infection**						
4	Single-strain	+	-	-	+	-
5	Multi-strain	+	+	+	+	-
6	ETEC control	-	-	-	+	-

+ and–indicate with or without probiotic supplementation, antibiotic administration or ETEC infection.

Following weaning at 21 days of age, pigs in groups 1–3 were not challenged with ETEC, but received 3 ml of sterile peptone water (Becton, Dickinson and Company, Maryland, USA) at the same time that the ETEC groups (groups 4–6) were challenged. Piglets in the negative control group (group 1) were fed with a basal diet without probiotic and antibiotics. Piglets in the probiotic control group (group 2) were orally supplemented with a 3 ml double-coated multi-strain LAB mixture (L22F, L25F, and P72N) suspended in sterile peptone water at a final concentration at 10^9^ CFU/ml through sterile syringe, receiving this on the five occasions mentioned above. Following weaning, piglets in the antibiotic group (group 3) were fed with a basal diet mixed with antibiotic (chlortetracycline at 300mg/kg), as previously described [20].

In the ETEC challenged groups (groups 4–6), piglets in all groups were fed with a basal diet after weaning. Those in the single strain group (group 4) as neonates previously had been orally supplemented with 3 ml of double-coated single-strain LAB (P72N) suspended in sterile peptone water at a final concentration at 10^9^ CFU/ml via sterile syringe, whilst those in the multi-strain group (group 5) had been orally supplemented with 3 ml of double-coated multi-strain LAB mixture (L22F, L25F, and P72N) suspended in sterile peptone water at a final concentration at 10^9^ CFU/ml through sterile syringe. The piglets in the ETEC control group (group 6) only received 3 ml of sterile peptone water. All piglets in the three ETEC challenged group were orally inoculated with ETEC strain L3.2 at a final concentration at 5×10^9^ CFU/ml at 28 days of age (7 days after weaning).

Faeces samples were obtained from individual piglets through digital stimulation of the rectum. Approximately five grams of faeces were collected from four of the piglets (2 male and 2 female) in each group on Day 2, 12 hours post-challenge (hpc) and 14 days post-challenge (dpc), with different pigs sampled at each collection. For each group and each collection time, the four faecal samples were combined into one pooled sample before genomic DNA extraction. Faeces were collected into sterile containers and stored at -20˚C until processed within a week of collection.

### DNA extraction and shotgun metagenomic sequencing

Total genomic DNA was extracted from each pooled faecal sample from four piglets per treatment per timepoint using the Quick-DNA/soil microbe microprep kit (Zymoresearch, CA, USA) according to the manufacturer’s recommendation. The extracted DNA was checked for purity by *A*_*260*_/*A*_*280*_ comparison using the OneDrop TOUCH lite micro-volume spectrophotometer (Biometrics Technologies, Wilmington, DE, USA). DNA degradation was checked by 2% agarose gel electrophoresis (Vivantis, Selangor Darul Ehsan, Malaysia) and visualized under UV in the Syngene™ Ingenius 3 Manual Gel Documentation System (SynGene InGenius, Cambridge, UK). In addition, the total DNA concentration was measured using a Qubit™ 4 fluorometer with the dsDNA broad-range assay kit (Invitrogen™, Thermo Fisher Scientific, Waltham, USA). Shotgun metagenomic sequencing was undertaken using the Illumina Novaseq 6000 on the Illumina HiSeq-PE150 platform at 10-GB data output according to the manufacturer’s instructions (Novogene Bioinformatics Technology Co. Ltd., Beijing, China).

### Quality control

The paired-end raw sequence reads were quality filtered in several steps for removing sequencing adapters and low-quality sequences with quality scores <30 using Trimmomatic v.0.36.5 [23]. Finally, any sequences mapped to the pig genome (*Sus scrofa*, NCBI accession no. NC010443) were filtered out using Bowtie2 v.2.3.4.32 [24]. All the bioinformatic analyses were performed on the European Galaxy server (https://usegalaxy.eu/).

### Taxonomic annotation

The taxonomic classifications of the metagenome datasets were identified by Kraken2 (Galaxy Version 2.0.85) (k = 35, ℓ = 31). The Kraken2 database, the complete genomes in RefSeq for the bacterial, archaeal, and viral domains, the human genome and a collection of known vectors were all retrieved from NCBI [25]. Alpha diversity (Species richness, Shanon and Simpson diversity index) and beta-diversity (Bray-Curtis dissimilarity matrix) were analyzed with the QIIME2 platform version 2021.4 (https://qiime2.org/) [26].

### Antibiotic resistance, metal resistance and biocide resistance gene annotation

The clean raw reads after the quality filtering processes were used for similarity searches against the antimicrobial resistance, metal resistance and biocide resistance MEGARes database [27] by using NCBI BLAST+ blastn (Galaxy Version 2.10.1) [28]. The MEGARes database that contains the sequences of approximately 7,868 nucleotide sequences of antimicrobial resistance genes (ARGs) based on a nonredundant compilation of sequences contained in ResFinder, ARG-ANNOT, the Comprehensive Antibiotic Resistance Database (CARD, the National Center for Biotechnology Information (NCBI) Lahey Clinic beta-lactamase archive and BacMet was accessed on 14-10-2019.

### MGEs annotation

The clean raw reads after the quality filtering processes were used for similarity searches for plasmids using the PlasmidFinder database [29] and for class 1, 2, and 3 integron integrase genes in the INTEGRALL database [30, 31] by using NCBI BLAST+ blastn (Galaxy Version 2.10.1) [28]. The PlasmidFinder database contains approximately 469 nucleotide sequences accessed on 13-07-2020, whereas the INTEGRALL database contains 11 nucleotide sequences related to class 1, 2, and 3 integron integrase genes. After the quality filtering processes, the clean raw reads were used for similarity searches against insertion sequences in the ISFinder database [32] by using Diamond (Galaxy Version 0.9.21.0) [33]. The ISFinder database contains approximately 8,836 amino acid sequences and was accessed on 6-10-2020.

Additionally, the confidence match to those databases associated with antibiotic resistance genes and mobile genetic elements was set by considering both percent identity cutoff at 90% and minimum query coverage at 80%, as suggested elsewhere [8, 31]. Moreover, the results of taxonomic profiles, antibiotic resistance, and mobile genetic elements were illustrated in the form of relative abundance by the total count method, which was performed as previously described [34].

### Functional annotation

The clean raw reads from each sample were *de novo* metagenomic assembled with default settings using MEGAHIT (Galaxy Version 1.1.3.43) [35]. The assembled contigs were examined for genome assembly quality using Quast (Galaxy Version 5.0.24) [36]. Functional annotation was determined through metagenome rapid annotation using subsystem technology server version 4 (MG-RAST) [37]. The assembled contigs were submitted to MG-RAST and functional annotation, and they were performed against the Kyoto Encyclopedia of Genes and Genomes database (KEGG) database for analyzing metabolism and SEED subsystem database for analyzing stress response which applied the following thresholds: >60% identity, 15 amino acids for a minimum alignment length, and e-value <1e-5. The investigated markers of stress response were catalase, fumarate and nitrate reduction regulatory protein, iron-binding ferritin-like antioxidant protein, redox-sensitive transcriptional regulator, superoxide dismutase and transcriptional regulator. In addition, the functional results were presented in normalized abundance which was generated by MG-RAST using DESeq analysis, as suggested elsewhere [2].

## Results

### Overall sequencing data and microbial diversity of the piglet faecal samples

DNA extracted from piglet faeces was sequenced with Illumina Hi-seq, obtaining 1.4 billion reads with read counts ranging from 68.9 to 115.2 million. After quality filtering, 1.2 billion high-quality readings were acquired, resulting in an 89.47 percent clean-read rate **(S2 Table)**. After *de novo* metagenomic assembly by MEGAHIT, there were 133,927 to 624,196 assembled contigs **(S3 Table)**. The species richness and diversities (Shanon and Simpson) of gut microbial alpha diversity were lower in the probiotic control group than in the negative control and antibiotic groups within the non-ETEC challenged groups at 12-hours and 14-days after the time of ETEC challenge. However, amongst the ETEC challenged groups, the multi-strain group tended to have greater alpha diversity than the single-strain and ETEC control groups, in terms of both species richness and diversity **(S4 Table)**. The principal coordinate analysis (PCoA) plot on Day 2 (two days of age; before probiotic treatment), at hour 12 (12-hour post-ETEC infection, 12 hpc), and at day 42 (14 days post-ETEC infection, 14 dpc) demonstrated three different clusters, as shown in **S2 Fig**.

### Taxonomic abundance and composition of the piglet gut microbiota

**Fig 1:**
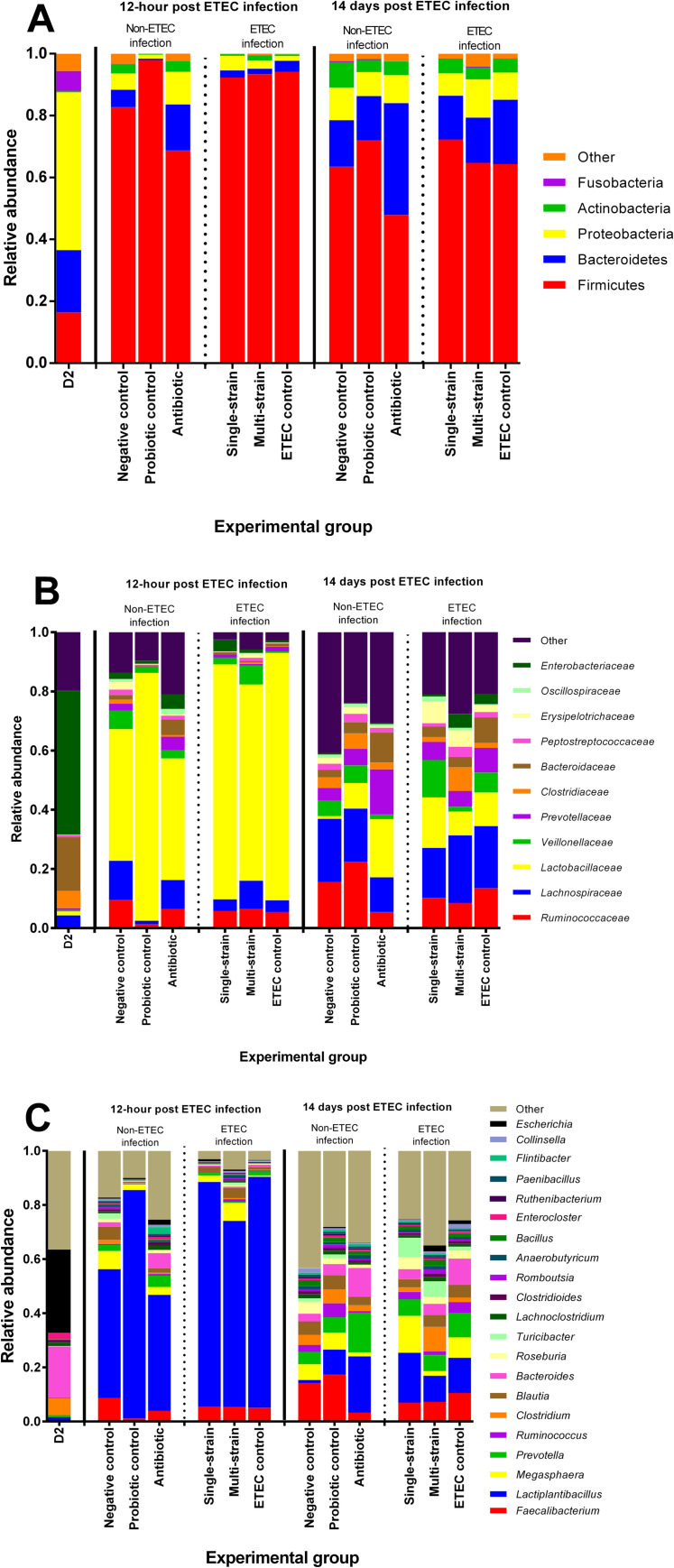
The relative abundance of faecal taxonomic classification across treatments in each time-point at the phylum (A), family (B), and genus levels (C), respectively, according to annotation with the Kraken2 database. D2 refers to 2 days of age, before probiotic treatment.

The abundance and composition of bacterial taxonomic groups at the phylum, family, and genus level are depicted in **Fig 1**. The most prevalent phyla at 2 days of age (Day 2) were Proteobacteria and Bacteroidetes (**Fig 1A**). The two most common families that were identified were *Enterobacteriaceae* and *Bacteroidaceae* (**Fig 1B**). Furthermore, at Day 2, piglet faeces samples were enriched in the genera *Escherichia* and *Bacteroides* (**Fig 1C**).

The average relative abundance of the Firmicutes, Bacteroidetes, and Proteobacteria phyla was approximately 97% of the total abundance at 12 hpc **(Fig 1A)**. In the non-ETEC infected groups, the probiotic control group had a higher proportion of members of the Firmicutes phylum and *Lactobacillaceae* family, while Proteobacteria were found in the highest abundance in the antibiotic group (**Fig 1A and 1B**). The antibiotic group had a higher percentage of *Bacteroidaceae* than the other groups (**Fig 1B**). Furthermore, the probiotic control group had an increased quantity of *Lactiplantibacillus* genus (**Fig 1C**). In the ETEC challenged groups, Firmicutes were found to be the most abundant in all experimental groups, at more than 92% (**Fig 1A**). Firmicutes phylum members *Lachnospiraceae*, *Veillonellaceae* and *Ruminococcaceae* were increased in the multi-strain group (**Fig 1B**). In addition, when compared to the single-strain and ETEC control groups, the relative abundance of *Megasphaera*, *Blautia* and *Ruminococcus* was higher in the multi-strain group (**Fig 1C**).

At 14 dpc, the dominating phyla showed a similar trend as at 12 hpc, with Firmicutes, Bacteroidetes, and Proteobacteria enriched across the experimental groups **(Fig 1A)**. In the non-ETEC infected groups, members of the Firmicutes phylum and *Ruminococcaceae* family were found in greater abundance in the probiotic control group than in the other groups, while the *Bacteroidetes* phylum and *Bacteroidaceae* family were still prominent in the antibiotic group **(Fig 1A and 1B)**. Furthermore, piglets in the probiotic control group showed higher levels of the genera *Faecalibacterium*, *Megasphaera* and *Ruminococcus* (**Fig 1C**). All the ETEC challenged groups exhibited a high proportion of members of the Firmicutes phylum **(Fig 1A)**. At the family level, *Lachnospiraceae* and *Clostridiaceae* were markedly increased in the multi-strain group. In contrast, a high abundance of *Bacteroidaceae* also was observed in the ETEC control group **(Fig 1B)**. Furthermore, the genera *Clostridium* and *Bacillus* were enriched in the multi-strain group. At the same time, the ETEC control group had a higher number of *Bacteroides* genus than the other groups **(Fig 1C)**.

### Abundance and composition of the piglet gut resistome

**Fig 2:**
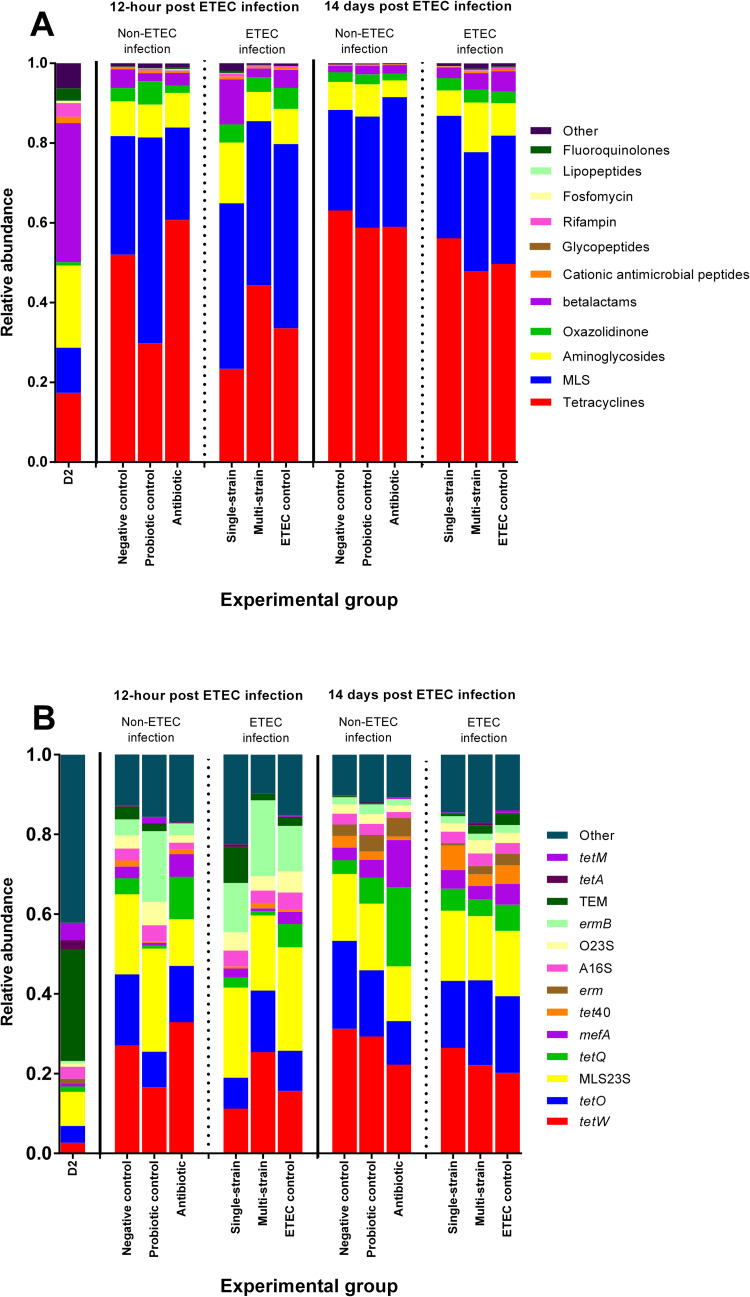
The relative abundance distribution of faecal antimicrobial resistance classes (A) and groups (B) across treatments at each time-point based on annotation with MEGARes database. D2 refers to 2 days of age, before probiotic treatment.

At Day 2, TEM genes associated with beta-lactam resistance were the most prominent antimicrobial resistance (AMR) determinants (**Fig 2A and 2B**). The beta-lactam resistance class was enriched in the negative control and antibiotic groups of the non-ETEC infected groups at 12 hpc (**Fig 2A**). In addition, the *tetW* and *tetQ* genes were overrepresented in those groups (**Fig 2B**). Beta-lactam resistance in the ETEC challenged groups was lower in the multi-strain group than in the single-strain and ETEC control groups (**Fig 2A)**. Furthermore, the single-strain and ETEC control groups had more TEM and *tetQ* genes than the multi-strain group (**Fig 2B**).

At 14 dpc, amongst the non-ETEC infected groups beta-lactam resistance was dominant in the antibiotic group **(Fig 2A)**. In the antibiotic group, the *tetQ*, *mefA* and *tetM* genes were all found in abundance **(Fig 2B)**. Furthermore, in the ETEC challenged groups, the *tetQ*, *mefA* and *tetM* genes were less frequent in the multi-strain group than in the single-strain and ETEC control groups **(Fig 2A and 2B)**.

### Abundance and diversity of metal and biocide resistance

According to the metal resistance analysis, multi-metal resistance was the most common type identified, followed by copper (Cu) and zinc (Zn) resistance (**Table 2**). At 12 hpc and 14 hpc, the Cu and Zn resistances were more abundant in the antibiotic group than in the negative control and the probiotic control groups. Moreover, Cu resistance in the single-strain group was higher than in the multi-strain and the ETEC control groups **(Table 2)**.

**Table 2:** The percentage of relative abundance of metal resistance group based on metal resistance genes in piglet faecal samples.

Metal resistance group	D2	12-hours post ETEC challenging						14-days post ETEC challenging					
		Non-ETEC infection			ETEC infection			Non-ETEC infection			ETEC infection		
		Negative control	Probiotic control	Antibiotic	Single-strain	Multi-strain	ETEC control	Negative control	Probiotic control	Antibiotic	Single-strain	Multi-strain	ETEC control
Multi-metal	57.11	67.27	67.50	59.07	47.83	66.15	55.56	55.62	61.78	57.14	60.77	56.79	55.88
Copper	13.31	10.12	8.93	14.65	35.75	10.76	12.59	11.23	15.53	42.86	12.10	12.00	19.80
Nickel	8.96	7.00	7.02	8.22	5.37	6.31	9.35	7.40	7.05	0.00	9.86	9.24	7.98
Zinc	8.52	6.38	6.84	7.77	4.17	7.17	9.45	12.05	7.38	0.00	6.57	8.42	7.54
Arsenic	5.41	5.65	4.91	5.36	3.36	5.42	5.59	6.58	4.96	0.00	6.64	6.89	4.90
Sodium	4.93	3.15	3.80	4.22	2.98	3.63	5.25	1.92	2.64	0.00	3.08	5.12	3.27
Iron	0.66	0.02	0.23	0.03	0.01	0.12	0.02	1.64	0.00	0.00	0.00	0.00	0.00
Chromium	0.62	0.41	0.54	0.43	0.50	0.41	0.64	1.10	0.00	0.00	0.56	0.62	0.49
Mercury	0.46	0.00	0.20	0.00	0.01	0.03	0.28	2.47	0.66	0.00	0.00	0.00	0.15
Tellurium	0.01	0.00	0.03	0.25	0.01	0.00	1.27	0.00	0.00	0.00	0.42	0.91	0.00

D2 refers to 2 days of age, before probiotic treatment.

Multi-biocide resistance was the most common biocide resistance, followed by acid and acetate resistance. At 12 hpc and 14 hpc, amongst the non-ETEC infected groups the multi-biocide resistance in the probiotic control group was lower than in the negative control and antibiotic groups. The multi-strain group had lower multi-biocide resistance and more abundant peroxide resistance than the single-strain and ETEC control groups in the ETEC infection groups (**Table 3**).

**Table 3:** The percentage of relative abundance of metal resistance group based on biocide resistance genes in piglet faecal samples.

Biocide resistance group	D2	12-hours post ETEC challenging						14-days post ETEC challenging					
		Non-ETEC infection			ETEC infection			Non-ETEC infection			ETEC infection		
		Negative control	Probiotic control	Antibiotic	Single-strain	Multi-strain	ETEC control	Negative control	Probiotic control	Antibiotic	Single-strain	Multi-strain	ETEC control
Acid	34.427	32.567	29.961	37.233	31.562	32.426	32.642	27.723	35.223	0	29.978	30.665	30.196
Multi-biocide	34.392	34.602	26.800	32.058	37.785	33.557	36.889	38.614	27.935	100	40.940	36.569	37.444
Acetate	18.453	18.394	20.185	14.820	16.721	17.470	19.358	27.723	12.955	0	16.555	21.628	18.806
Peroxide	8.905	10.969	9.922	11.755	10.628	13.322	7.556	3.960	21.053	0	11.186	7.948	10.655
Phenolic compound	3.799	3.468	3.210	4.134	3.304	3.226	3.556	1.980	2.834	0	1.342	3.191	2.899
Biguanide	0.007	0	0	0	0	0	0	0	0	0	0	0	0
Quaternary ammonium compounds	0.016	0	9.922	0	0	0	0	0	0	0	0	0	0
Paraquat	0.001	0	0	0	0	0	0	0	0	0	0	0	0

D2 refers to 2 days of age, before probiotic treatment.

### Mobile genetic elements (plasmid replicons, integron integrase genes and insertion sequences) within the piglet gut microbial community

**Fig 3:**
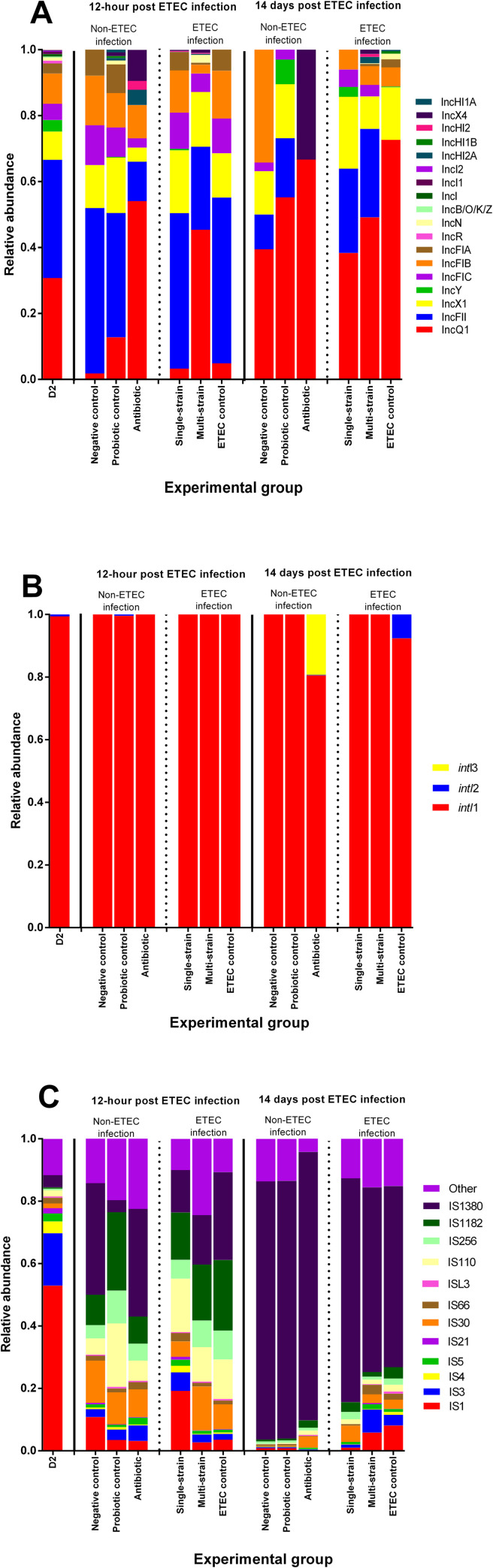
Relative abundance of genes based on mobile genetic element annotation across treatments in each time-point. Stacked bar plot demonstrating the relative abundance distribution of classified plasmid replicons (A). Stacked bar plot illustrating the relative abundance distribution of aligned integron integrase genes (B). Stacked bar plot displaying the relative abundance distribution of sorted insertion sequences (C). D2 refers to 2 days of age, before probiotic treatment.

The antibiotic group had higher levels of several plasmid replicons, including IncQ1, IncX4, IncHI2, and IncHI2A than the other groups (**Fig 3A**). Integrase gene (*intI*) class 1 was the most common integron in all experimental groups, accounting for more than 97% of all detected integrons. Furthermore, an *intI* class 2 was found in the ETEC control group at 14 dpc, whereas an *intl* class 3 was only found in the antibiotic group (**Fig 3B**). At 12 hpc and 14 dpc, insertion sequence (IS) 1380 was enriched in the negative control and antibiotic groups (**Fig 3C**). IS1380 was prominently detected in the ETEC infected groups, while IS3 and IS30 were prominently detected in the single-strain and multi-strain groups (**Fig 3C**).

### Microbial functional diversity of the gut metagenome related to stress response in ETEC and non-ETEC infected piglets

**Fig 4:**
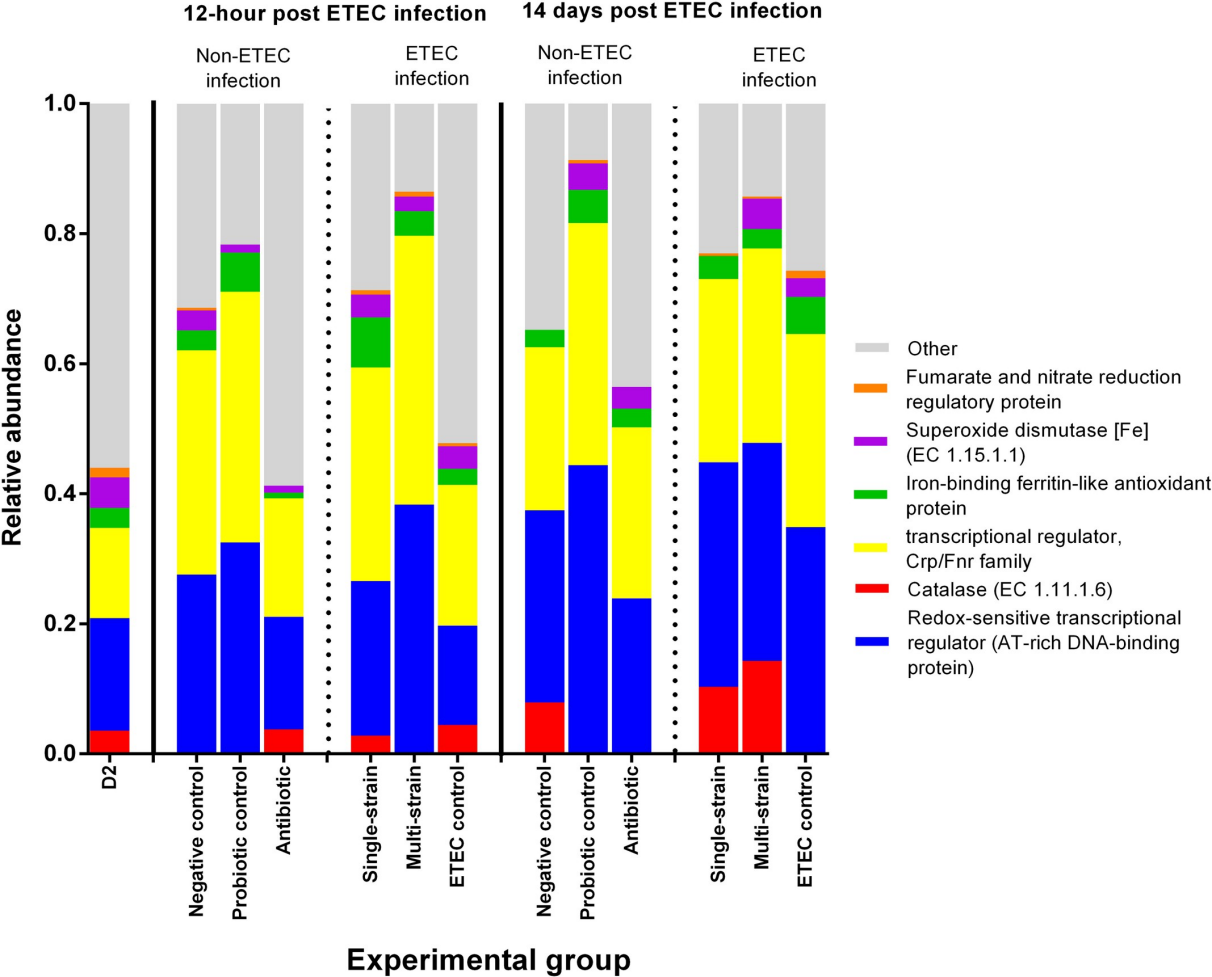
Relative abundance of the level 4 SEED subsystem classified reads associated with oxidative stress from piglet faecal samples in ETEC or non-ETEC infected piglets. D2 refers to 2 days of age, before probiotic treatment.

The results for the stress response that was analyzed using the SEED subsystem database within the MG-RAST server are summarised in **S3 Fig** and **Fig 4**. In all experimental groups, oxidative stress was the most prevalent response, ranging from 33.20 to 45.63% in the stress response at level 2. Surprisingly, the multi-strain group had the highest stress response associated with detoxification at 12 hpc, accounting for more than 19% of the total (**S3 Fig**). The transcriptional and redox-sensitive transcriptional regulators, which were the main markers of oxidative stress responses in this study, were found in approximately 80% of the total sequences in the probiotic control and multi-strain groups. In addition, compared to the other groups, the multi-strain group had more catalase and superoxide dismutase (**Fig 4**).

### Microbial functional diversity of the gut metagenome associated with nutrient metabolism in ETEC and non-ETEC infected piglets

The relative abundance of functional genes at level 1 KEGG related to metabolism is shown in **S4 Fig**. Amino acid and carbohydrate metabolism were dominant in roughly 60% of the total nutrient metabolism sequences (**S4 Fig**). Most amino acid metabolism pathways involved alanine, aspartate, and glutamate metabolism, followed by glycine, serine and threonine metabolism, and cysteine and methionine metabolism (**Table 4**). Furthermore, glycolysis/gluconeogenesis, amino sugar and nucleotide sugar metabolism, and galactose metabolism were the top three carbohydrate metabolisms, respectively (**Table 5**). Among the non-ETEC infected groups, amino acid and carbohydrate metabolism pathways were less represented in the probiotic control group than in the antibiotic group at 12 hpc. The multi-strain group, on the other hand, had more genes related to amino acid metabolism than did the other ETEC infected groups **(Table 4)**. The probiotic control and multi-strain groups had more genes associated with amino acid and carbohydrate metabolism at 14 dpc than the other groups **(Tables 3 and 4)**.

**Table 4:** Normalized abundance of the level 3 KEGG functional reads related to amino acid metabolism from faecal samples in ETEC or non-ETEC challenging piglets.

Amino acid metabolism	D2	12-hours post ETEC challenging						14-days post ETEC challenging					
		Non-ETEC infection			ETEC infection			Non-ETEC infection			ETEC infection		
		Negative control	Probiotic control	Antibiotic	Single-strain	Multi-strain	ETEC control	Negative control	Probiotic control	Antibiotic	Single-strain	Multi-strain	ETEC control
Glycine, serine, and threonine metabolism	1310	1144	3799	5668	2054	2732	2004	3382	3715	2852	4428	4668	2511
Alanine, aspartate, and glutamate metabolism	1390	1576	4677	6664	2775	3241	2511	4107	4512	3936	5647	5694	2004
Arginine and proline metabolism	837	792	2405	3453	1377	1767	1239	2213	2369	1914	2751	2948	1918
Cysteine and methionine metabolism	825	1018	3487	4642	1933	2521	1918	3135	3188	2629	3843	4213	1239
Lysine biosynthesis	639	740	2136	2937	1222	1505	1131	2011	2160	1573	2557	2761	1131
Phenylalanine, tyrosine, and tryptophan biosynthesis	504	515	1648	2320	1014	1129	909	1549	1667	1241	2029	1975	966
Valine, leucine, and isoleucine biosynthesis	494	444	1446	2191	965	1108	966	1515	1596	1268	1779	2018	919
Histidine metabolism	501	542	1493	2222	847	1040	919	1435	1595	1226	1835	1914	909
Valine, leucine, and isoleucine degradation	328	194	626	1146	334	460	314	569	648	525	775	813	314
Phenylalanine metabolism	300	109	430	566	193	303	173	375	424	249	407	497	173
Tyrosine metabolism	194	157	352	427	186	284	163	341	345	209	401	469	163
Lysine degradation	129	36	121	191	54	94	42	94	127	93	129	160	42
Tryptophan metabolism	41	23	50	95	14	41	21	35	38	40	57	72	21

D2 refers to 2 days of age, before probiotic treatment.

**Table 5:** Normalized abundance of the level 3 KEGG functional reads associated with carbohydrate metabolism from faecal samples in ETEC or non-ETEC infected piglets.

Carbohydrate metabolism	D2	12-hours post ETEC challenging						14-days post ETEC challenging					
		Non-ETEC infection			ETEC -infection			Non-ETEC infection			ETEC infection		
		Negative control	Probiotic control	Antibiotic	Single-strain	Multi-strain	ETEC control	Negative control	Probiotic control	Antibiotic	Single-strain	Multi-strain	ETEC control
Glycolysis / Gluconeogenesis	880	830	2373	3202	1250	1652	1209	2194	2506	1609	2773	2980	2094
Pyruvate metabolism	848	692	1801	2463	975	1243	977	1757	1937	1276	2201	2218	1655
Amino and nucleotide sugar metabolism	712	684	2274	3037	1245	1464	1136	1961	2213	1413	2593	2731	1994
Galactose metabolism	748	784	2134	2668	1329	1576	1171	1878	2057	1627	2384	2631	2006
Pentose phosphate pathway	703	632	1769	2377	923	1338	866	1602	1858	1162	2133	2152	1563
Starch and sucrose metabolism	569	673	1892	2653	1088	1493	1061	1679	1836	1458	2259	2382	1764
Fructose and mannose metabolism	567	535	1457	2360	894	966	820	1201	1337	1204	1742	1785	1289
Pentose and glucuronate interconversions	554	515	1392	1948	863	990	784	1048	1424	1182	1548	1507	1295
Citrate cycle (TCA cycle)	318	330	1146	1805	562	729	520	1031	1065	941	1362	1408	888
Glyoxylate and dicarboxylate metabolism	224	158	592	724	318	416	253	616	643	380	641	648	473
Ascorbate and aldarate metabolism	92	30	62	68	45	52	34	62	63	59	46	72	66
Butanoate metabolism	80	74	256	347	104	188	120	206	238	188	270	285	204
Inositol phosphate metabolism	60	28	103	106	38	77	43	82	90	42	90	117	71
Propanoate metabolism	58	21	69	80	35	36	36	43	63	34	75	65	44
C5-Branched dibasic acid metabolism	12	0	21	31	0	25	3	26	34	11	18	37	15

D2 refers to 2 days of age, before probiotic treatment.

## Discussion

In our previous study that used 60 of the pigs included in the current study, dosing the neonatal piglets with the multi-strain probiotic enhanced average daily gain and feed conversion ratio (FCR) of the piglets after ETEC challenge following weaning, whilst supplementing with the single-strain probiotic increased FCR [22]. The piglets receiving probiotics had an increase in lactic acid bacteria counts and a decrease in *E*. *coli* counts in the faeces, with lower levels of virulence genes being detected. Challenged piglets receiving probiotics had milder intestinal lesions with better morphology, including greater villous heights and villous height per crypt depth ratios, than pigs just receiving ETEC. This study demonstrated that prophylactic administration of microencapsulated probiotic strains may improve outcomes in weaned pigs with colibacillosis. The current study enlarged on these findings by examining the gut microbiota of these pigs in more detail. An additional group of pigs receiving chlortetracycline after weaning was included to help compare probiotics and antimicrobials in influencing the gut microbiota and supporting pig health after weaning. Tetracyclines are commonly given to piglets after weaning to help prevent the development of post-weaning diarrhoea. Whole-genome shotgun metagenomic sequencing of DNA extracted from faeces was used to investigate the gut microbiome, resistome, stress responses, and nutrient metabolism, and to examine how the probiotics cause beneficial changes in piglets infected with ETEC.

Faecal samples were used as a proxy for intestinal samples for examining the gut microbiota, as faeces can be obtained from live pigs which then can be sampled again at later stages. The gut microbiota composition in faeces collected from the rectum seems to be stable, and it shows the same pattern as the hindgut regions, indicating that the faecal microbiota can be used as a proxy for the microbiota in the large intestine of the pigs [38, 39]. Samples were pooled because it was not technically or financially possible to examine samples from all individual piglets in this study. It is acknowledged that this does not allow comparison of variations between pigs within a group, but this approach was necessary for practical purposes and does provide an overview of group affects. The methodology used means that it was not appropriate to undertake statistical analysis between groups in this study.

Faecal microbial diversity expanded over time during the weaning period, which was consistent with previous findings [8, 40]. Firmicutes and Proteobacteria were the most prevalent phyla found in piglet faeces at Day 2, which agrees with a previous study which found that these phyla were the most prevalent microbial components in early newborn piglets [41]. In addition, the genus *Escherichia*, which belongs to the *Enterobacteriaceae* family, was found in abundance. Pathogenic strains of *Escherichia coli* can have an impact on human and animal health by acquiring and disseminating AMR and virulence genes through the food supply chain, and they act as a biomarker for piglets that may develop diarrhoea in the lactation phase [4, 42].

According to several studies, Firmicutes and Bacteroidetes are the most numerous phyla in the piglet faecal microbiota during the post-weaning phase [4, 8, 13, 40]. The probiotic control group had a larger proportion of the Firmicutes phylum than the other groups in the current study. This result appears to be congruent with another study, which found that supplementing with *Enterococcus faecalis* UC-100 was associated with more than 85% of the total sequences enriched by the Firmicutes phylum [40]. In the current study the genera *Blautia*, *Lactiplantibacillus*, *Megasphaera*, *Ruminococcus*, *Clostridium* and *Faecalibacterium* were identified in the multi-strain and probiotic control groups. These genera are regarded as being beneficial microbes due to a variety of characteristics, including the ability to produce antibacterial substances (e.g., bacteriocins, organic acids) that inhibit growth of pathogens, the ability to increase carbohydrate metabolism by utilizing dietary starch and fiber, and the ability to produce short-chain fatty acids (SCFAs) that reduce gut inflammation [2, 7, 13, 40, 43–46]. The group receiving chlortetracycline showed an increase in Proteobacteria and Bacteroidetes, which is consistent with prior research demonstrating that antibiotic administration could boost these phyla [6–8, 47]. However, several studies have suggested that enhanced numbers of Bacteroidetes may promote host health by enhancing nutrient digestion and absorption [2, 7]. Furthermore, it has been suggested that they act as a biomarker for gut dysbiosis in piglets given antibiotics [48].

We found a variety of AMR determinants in neonatal piglets in this study, and the dominant antibiotic-resistant classes and genes discovered in this study appear to be linked to our previous research, which found that neonatal piglets in antibiotic-free farms had high levels of beta-lactam resistance and carriage of the *blaTEM* gene [49]. The World Health Organization classifies beta-lactams as critically important antimicrobials, meaning they have the potential to have a major impact on human health [50].

Previous studies have shown that tetracyclines and MLSs are the most common antibiotic resistance classes in weaned pigs receiving or not receiving in-feed antibiotics, and the findings of the current study are consistent with this [8, 47]. High levels of beta-lactam resistance also were found in both the negative control and the antibiotic groups. This matches previous findings of dominant beta-lactam resistance in medicated and unmedicated piglets [8]. The antibiotic group had more *tetW*, *tetQ*, *tetM* and *mefA* genes, which are involved in tetracycline ribosomal protection proteins and MLS efflux pumps, on an AMR gene level [8, 51]. These genes have been found on mobile genetic elements such as conjugative transposons, which can spread to other bacteria via horizontal transfer. Furthermore, previous research has found that the *Bacteroidaceae* family frequently carry such genes, suggesting that they could be a source of AMR genes for the gut microbial community [8, 52].

Piglets given probiotics in the current study had a lower proportion of AMR determinants like beta-lactam resistance, *mefA*, *tetQ*, and *tetW* genes than piglets given antibiotics. Probiotics may modify the gut microbial population by reducing the abundance of some antibiotic-resistant microorganisms through a variety of processes, including competition for food substrates and binding sites, production of antimicrobial compounds, and regulation of immune responses [12]. These data are consistent with prior research showing that probiotic treatment in infants can reduce ARG abundance by eliminating antibiotic-resistant carriers [14]. To our knowledge, this is the first study to investigate the effects of probiotic supplementation on modulation of the pig gut resistome. However, since the existence of some antibiotic genes may not indicate phenotypic resistance, a weakness in the current study was the lack of comparison between AMR genotypic and phenotypic features. Phenotypic determinations should be performed on fresh faecal samples, and this was not possible with the frozen samples [6, 47].

Based on co-selection processes such as co-resistance, cross-resistance, and biofilm formation, there is evidence of a link between antibiotic, metal, and biocide resistances [53]. Copper and multi-biocide resistances were found in abundance in the antibiotic group, which was linked to numerous antimicrobial drug resistances such as to beta-lactams, fluoroquinolones, macrolides and tetracyclines [54–56]. This could explain why the antibiotic group had more Cu and multi-biocide resistance than the other groups. Biofilm production is critical for preserving metal and biocide resistances, protecting the population from metal and biocide toxicity, and increasing the lateral transfer of ARGs with co-selected metal resistant genes [53, 55]. Our probiotic strains have been shown to minimize ARG transfer and biofilm development *in vitro* [17]. Taken together, this could be another reason why the probiotic supplemented groups had lower Cu and multi-biocide resistance genes detected.

The complete set of MGEs, and specifically the mobilome, are thought to hasten the spread of ARGs among members of the gut microbiota [57]. In the antibiotic group, IncQ1, IncX4, IncHI2, and IncHI2A plasmids were detected in abundance, which is of concern because it may allow multidrug resistance (MDR) in humans and animals, such as resistance to aminoglycosides, beta-lactams, and tetracycline [58]. Furthermore, they may be involved in colistin resistance where they contain the mobilized colistin resistance (*mcr*) gene [58, 59]. Interestingly, the probiotic-supplemented groups had fewer plasmid replicons than the antibiotic-supplemented group. This finding supports the theory that probiotics can regulate the gut microbial community by lowering the proportion of microbiota carrying certain plasmids, or by blocking ARG transfer via a variety of pathways [12, 17]. In the current study, class 1 integrons were shown to be abundant in all groups. This finding is consistent with prior research that found it to be the most common integron type, accounting for about 80% of all types in enteric bacteria in humans and animals [60]. The ETEC control group contained class 2 integron, which is involved in resistance to aminoglycosides, beta-lactams, and erythromycins [60, 61]. In addition, class 3 integron was found only in the antibiotic group, and it has been linked to beta-lactam resistance and the IncQ plasmid replicon [60]. Furthermore, the antibiotic group had higher levels of IS1380, which can increase beta-lactam and nitroimidazole resistance in Bacteroidetes, the antibiotic group’s predominant member [62]. In our study, the probiotic supplemented groups had more IS3 and IS30, which are involve with numerous metabolic modulations such arginine production and the use of acetate, citrate, and galactose [62]. This appears to be the first report to detail the effects of probiotic supplementation on MGE regulation in the pig gut microbial population.

An imbalance between reactive oxygen species (ROS) and antioxidant responses was typically seen in the weaning transition or after ETEC infection, which events are likely to be a source of oxidative stress [2]. Excessive exposure to ROS can have negative consequences on bacterial cells, resulting in protein activity dysfunction and bacterial cell death [2]. In the probiotic groups, genes related to the oxidative response, particularly “transcriptional regulator” and “redox-sensitive transcriptional regulator” contributing to antioxidant activity, were elevated [63]. Furthermore, antioxidant capacity was related to detoxification in the multi-strain group following ETEC challenge [63]. This finding agrees with previous studies suggesting that a variety of probiotic isolates may boost antioxidant defense mechanisms and reduce oxidative stress [5, 64]. Consequently, further research on the antioxidant activities of our probiotic strains (L22F, L25F, and P72N) is needed to improve understanding of the mechanism of stress response modulation.

The probiotic groups had increased numbers of amino acid metabolism genes, which agrees with previous work showing that many bacterial species, including *Bifidobacterium*, *Lactiplantibacillus*, *Megasphaera* and *Veillonella* are involved in modulating amino acid metabolism [5, 65]. Moreover, several amino acids, including alanine, arginine, cysteine, glutamine, glycine, lysine, methionine and threonine have been shown to benefit pig gut health, including by altering the gut microbiota, maintaining intestinal shape, and increasing gut immunological, anti-inflammatory, and anti-oxidative stress functions [66]. We also found that the probiotic supplemented groups had higher levels of genes involved in glucose metabolism. This result is consistent with previous studies that identified carbohydrate utilization via fermentation and hydrolysis pathways was found in a variety of gut bacteria, including *Bifidobacterium*, *Faecalibacterium*, *Lactiplantibacillus* and *Ruminococcus*, [5, 65]. SCFAs, which are readily available energy sources for pigs, are one of the bacterial metabolites produced following food digestion that may have anti-inflammatory and antagonistic properties [13, 65]. However, additional investigations into the complete genomes of our probiotic strains are recommended to expand these findings. These data should be linked to global metabolomic and proteomic studies to better understand the mechanisms of the probiotic effects on the gut microbiome and resistome.

## Conclusion

In conclusion, supplementing neonatal piglets with our microencapsulated probiotics helped to improve the composition of the gut microbiota by increasing the numbers and proportions of beneficial bacteria. These probiotic effects continued after weaning and were associated with improved performance and an enhanced antioxidant response in piglets challenged with ETEC. The changes in the microbiota benefited the piglets in other ways, including by reducing the antibiotic resistome, metal resistance, biocide resistance, and number of MGEs. Additionally, by enriching genes associated with amino acid and carbohydrate metabolism, probiotics boosted the antioxidant response to reduce oxidative stress and promote improved nutritional utilization. Taken together, these data shed light on probiotic effects on the gut microbiome and on resistome regulation.

## Supporting information

S1 Fig Schematic of experimental design and sample collection. D indicates day after birth and hpc refers to hours post ETEC challenge.
(DOCX)

S2 Fig Principal coordinate analysis (PCoA) plot based on Bray-Curtis dissimilarity index of microbial taxonomic profile at the species level from piglet faecal samples across treatments in each time-point. The geometric shapes demonstrate the group of samples in each time-point. D2 refers to 2 days of age, before probiotic treatment.
(DOCX)

S3 Fig Relative abundance of the level 2 SEED subsystem aligned genes associated with stress response from piglet faecal samples in ETEC or non-ETEC infected piglets. D2 refers to 2 days of age, before probiotic treatment.
(DOCX)

S4 Fig The relative abundance of the level 1 KEGG functional genes related to metabolism from piglet faecal samples in ETEC or non-ETEC infected piglets. D2 refers to 2 days of age, before probiotic treatment.
(DOCX)

S1 Table Ingredient composition and nutrient concentration of the experimental basal diet. (DOCX)

S2 Table Summary of overall sequencing data. (DOCX)

S3 Table The summary of all *de novo* assembled metagenomic sequence data by using MEGAHIT and determining by QUAST. (DOCX)

S4 Table Alpha diversity of gut microbial communities from piglet fecal samples across treatments and each time-point. (DOCX)

## References

[1] Fouhse JM, Zijlstra RT, Willing BP. The role of gut microbiota in the health and disease of pigs. Animal Frontiers. 2016;6(3):30–6. doi: 10.2527/af.2016-0031

[2] Guevarra RB, Hong SH, Cho JH, Kim BR, Shin J, Lee JH, et al. The dynamics of the piglet gut microbiome during the weaning transition in association with health and nutrition. J Anim Sci Biotechnol. 2018;9:54. doi: 10.1186/s40104-018-0269-6 ; PubMed Central PMCID: PMC6065057. 30069307 PMC6065057

[3] Guevarra RB, Lee JH, Lee SH, Seok MJ, Kim DW, Kang BN, et al. Piglet gut microbial shifts early in life: causes and effects. J Anim Sci Biotechnol. 2019;10:1. Epub 2019/01/18. doi: 10.1186/s40104-018-0308-3 ; PubMed Central PMCID: PMC6330741. 30651985 PMC6330741

[4] Sun J, Du L, Li X, Zhong H, Ding Y, Liu Z, et al. Identification of the core bacteria in rectums of diarrheic and non-diarrheic piglets. Sci Rep. 2019;9(1):18675. Epub 2019/12/12. doi: 10.1038/s41598-019-55328-y ; PubMed Central PMCID: PMC6904459. 31822779 PMC6904459

[5] Liao SFF, Nyachoti M. Using probiotics to improve swine gut health and nutrient utilization. Anim Nutr. 2017;3(4):331–43. doi: 10.1016/j.aninu.2017.06.007 WOS:000434261000003. 29767089 PMC5941265

[6] Tunsagool P, Mhuantong W, Tangphatsornruang S, Am-In N, Chuanchuen R, Luangtongkum T, et al. Metagenomics of antimicrobial and heavy metal resistance in the cecal microbiome of fattening pigs raised without antibiotics. Appl Environ Microb. 2021;87(8). ARTN e02684-2010.1128/AEM.02684-20. WOS:000635350600009. doi: 10.1128/AEM.02684-20 33547058 PMC8091117

[7] Wang T, Teng K, Liu Y, Shi W, Zhang J, Dong E, et al. *Lactobacillus plantarum* PFM 105 promotes intestinal development through modulation of gut microbiota in weaning piglets. Front Microbiol. 2019;10:90. Epub 2019/02/26. doi: 10.3389/fmicb.2019.00090 ; PubMed Central PMCID: PMC6371750. 30804899 PMC6371750

[8] Ghanbari M, Klose V, Crispie F, Cotter PD. The dynamics of the antibiotic resistome in the feces of freshly weaned pigs following therapeutic administration of oxytetracycline. Sci Rep. 2019;9(1):4062. doi: 10.1038/s41598-019-40496-8 ; PubMed Central PMCID: PMC6411716. 30858509 PMC6411716

[9] Zhao Y, Su JQ, An XL, Huang FY, Rensing C, Brandt KK, et al. Feed additives shift gut microbiota and enrich antibiotic resistance in swine gut. Sci Total Environ. 2018;621:1224–32. Epub 2017/10/22. doi: 10.1016/j.scitotenv.2017.10.106 . 29054657

[10] Sirichokchatchawan W, Apiwatsiri P, Pupa P, Saenkankam I, Khine NO, Lekagul A, et al. Reducing the risk of transmission of critical antimicrobial resistance determinants from contaminated pork products to humans in South-East Asia. Front Microbiol. 2021;12:689015. Epub 2021/08/14. doi: 10.3389/fmicb.2021.689015 ; PubMed Central PMCID: PMC8353453. 34385984 PMC8353453

[11] Hossain MI, Sadekuzzaman M, Ha SD. Probiotics as potential alternative biocontrol agents in the agriculture and food industries: A review. Food Res Int. 2017;100(Pt 1):63–73. doi: 10.1016/j.foodres.2017.07.077 . 28873730

[12] O’Toole PW, Cooney JC. Probiotic bacteria influence the composition and function of the intestinal microbiota. Interdiscip Perspect Infect Dis. 2008;2008:175285. doi: 10.1155/2008/175285 ; PubMed Central PMCID: PMC2648622. 19277099 PMC2648622

[13] Shin D, Chang SY, Bogere P, Won K, Choi JY, Choi YJ, et al. Beneficial roles of probiotics on the modulation of gut microbiota and immune response in pigs. PLoS One. 2019;14(8):e0220843. doi: 10.1371/journal.pone.0220843 ; PubMed Central PMCID: PMC6713323. 31461453 PMC6713323

[14] Casaburi G, Duar RM, Vance DP, Mitchell R, Contreras L, Frese SA, et al. Early-life gut microbiome modulation reduces the abundance of antibiotic-resistant bacteria. Antimicrob Resist Infect Control. 2019;8:131. doi: 10.1186/s13756-019-0583-6 ; PubMed Central PMCID: PMC6693174. 31423298 PMC6693174

[15] Ma Z, Lee S, Jeong KC. Mitigating antibiotic resistance at the livestock-environment interface:a review. J Microbiol Biotechnol. 2019;29(11):1683–92. doi: 10.4014/jmb.1909.09030 . 31693837

[16] Sirichokchatchawan W, Tanasupawat S, Niyomtham W, Prapasarakul N. Identification and antimicrobial susceptibility of lactic acid bacteria from fecal samples of indigenous and commercial pigs. Thai J Vet Med. 2017;47(3):329–38. WOS:000413621800004.

[17] Apiwatsiri P, Pupa P, Yindee J, Niyomtham W, Sirichokchatchawan W, Lugsomya K, et al. Anticonjugation and antibiofilm evaluation of probiotic strains *Lactobacillus plantarum* 22F, 25F, and *Pediococcus acidilactici* 72N against *Escherichia coli* harboring *mcr*-1 gene. Front Vet Sci. 2021;8:614439. Epub 2021/06/29. doi: 10.3389/fvets.2021.614439 ; PubMed Central PMCID: PMC8225926. 34179153 PMC8225926

[18] Sirichokchatchawan W, Pupa P, Praechansri P, Am-In N, Tanasupawat S, Sonthayanon P, et al. Autochthonous lactic acid bacteria isolated from pig faeces in Thailand show probiotic properties and antibacterial activity against enteric pathogenic bacteria. Microb Pathog. 2018;119:208–15. doi: 10.1016/j.micpath.2018.04.031 . 29678738

[19] Sirichokchatchawan W, Temeeyasen G, Nilubol D, Prapasarakul N. Protective effects of cell-free supernatant and live lactic acid bacteria isolated from Thai pigs against a pandemic strain of porcine epidemic diarrhea virus. Probiotics Antimicrob Proteins. 2018;10(2):383–90. doi: 10.1007/s12602-017-9281-y . 28434154 PMC7091344

[20] Pupa P, Apiwatsiri P, Sirichokchatchawan W, Pirarat N, Maison T, Koontanatechanon A, et al. Use of *Lactobacillus plantarum* (strains 22F and 25F) and *Pediococcus acidilactici* (strain 72N) as replacements for antibiotic-growth promotants in pigs. Sci Rep. 2021;11(1):12028. Epub 2021/06/10. doi: 10.1038/s41598-021-91427-5 ; PubMed Central PMCID: PMC8187408. 34103574 PMC8187408

[21] Pupa P, Apiwatsiri P, Sirichokchatchawan W, Pirarat N, Muangsin N, Shah AA, et al. The efficacy of three double-microencapsulation methods for preservation of probiotic bacteria. Sci Rep. 2021;11(1):13753. Epub 2021/07/04. doi: 10.1038/s41598-021-93263-z ; PubMed Central PMCID: PMC8253736. 34215824 PMC8253736

[22] Pupa P, Apiwatsiri P, Sirichokchatchawan W, Pirarat N, Nedumpun T, Hampson DJ, et al. Microencapsulated probiotic *Lactiplantibacillus plantarum* and/or *Pediococcus acidilactici* strains ameliorate diarrhoea in piglets challenged with enterotoxigenic *Escherichia coli*. Sci rep. 2022. Forthcoming. doi: 10.1038/s41598-022-11340-3 35505092 PMC9065055

[23] Bolger AM, Lohse M, Usadel B. Trimmomatic: a flexible trimmer for Illumina sequence data. Bioinformatics. 2014;30(15):2114–20. doi: 10.1093/bioinformatics/btu170 ; PubMed Central PMCID: PMC4103590. 24695404 PMC4103590

[24] Langmead B, Salzberg SL. Fast gapped-read alignment with Bowtie 2. Nat Methods. 2012;9(4):357–9. doi: 10.1038/nmeth.1923 ; PubMed Central PMCID: PMC3322381. 22388286 PMC3322381

[25] Wood DE, Lu J, Langmead B. Improved metagenomic analysis with Kraken 2. Genome Biol. 2019;20(1):257. doi: 10.1186/s13059-019-1891-0 ; PubMed Central PMCID: PMC6883579. 31779668 PMC6883579

[26] Bolyen E, Rideout JR, Dillon MR, Bokulich NA, Abnet CC, Al-Ghalith GA, et al. Reproducible, interactive, scalable and extensible microbiome data science using QIIME 2. Nat Biotechnol. 2019;37(8):852–7. doi: 10.1038/s41587-019-0209-9 ; PubMed Central PMCID: PMC7015180. 31341288 PMC7015180

[27] Doster E, Lakin SM, Dean CJ, Wolfe C, Young JG, Boucher C, et al. MEGARes 2.0: a database for classification of antimicrobial drug, biocide and metal resistance determinants in metagenomic sequence data. Nucleic Acids Res. 2020;48(D1):D561–D9. doi: 10.1093/nar/gkz1010 ; PubMed Central PMCID: PMC7145535. 31722416 PMC7145535

[28] Cock PJ, Chilton JM, Gruning B, Johnson JE, Soranzo N. NCBI BLAST+ integrated into Galaxy. Gigascience. 2015;4:39. doi: 10.1186/s13742-015-0080-7 ; PubMed Central PMCID: PMC4557756. 26336600 PMC4557756

[29] Carattoli A, Zankari E, Garcia-Fernandez A, Voldby Larsen M, Lund O, Villa L, et al. In silico detection and typing of plasmids using PlasmidFinder and plasmid multilocus sequence typing. Antimicrob Agents Chemother. 2014;58(7):3895–903. doi: 10.1128/AAC.02412-14 ; PubMed Central PMCID: PMC4068535. 24777092 PMC4068535

[30] Moura A, Soares M, Pereira C, Leitao N, Henriques I, Correia A. INTEGRALL: a database and search engine for integrons, integrases and gene cassettes. Bioinformatics. 2009;25(8):1096–8. doi: 10.1093/bioinformatics/btp105 . 19228805

[31] Stalder T, Press MO, Sullivan S, Liachko I, Top EM. Linking the resistome and plasmidome to the microbiome. ISME J. 2019;13(10):2437–46. doi: 10.1038/s41396-019-0446-4 ; PubMed Central PMCID: PMC6776055. 31147603 PMC6776055

[32] Siguier P, Perochon J, Lestrade L, Mahillon J, Chandler M. ISfinder: the reference centre for bacterial insertion sequences. Nucleic Acids Res. 2006;34(Database issue):D32–6. doi: 10.1093/nar/gkj014 ; PubMed Central PMCID: PMC1347377. 16381877 PMC1347377

[33] Buchfink B, Xie C, Huson DH. Fast and sensitive protein alignment using DIAMOND. Nat Methods. 2015;12(1):59–60. doi: 10.1038/nmeth.3176 . 25402007

[34] Pereira MB, Wallroth M, Jonsson V, Kristiansson E. Comparison of normalization methods for the analysis of metagenomic gene abundance data. BMC Genomics. 2018;19(1):274. Epub 2018/04/22. doi: 10.1186/s12864-018-4637-6 ; PubMed Central PMCID: PMC5910605. 29678163 PMC5910605

[35] Li D, Liu CM, Luo R, Sadakane K, Lam TW. MEGAHIT: an ultra-fast single-node solution for large and complex metagenomics assembly via succinct de Bruijn graph. Bioinformatics. 2015;31(10):1674–6. doi: 10.1093/bioinformatics/btv033 . 25609793

[36] Gurevich A, Saveliev V, Vyahhi N, Tesler G. QUAST: quality assessment tool for genome assemblies. Bioinformatics. 2013;29(8):1072–5. doi: 10.1093/bioinformatics/btt086 ; PubMed Central PMCID: PMC3624806. 23422339 PMC3624806

[37] Meyer F, Bagchi S, Chaterji S, Gerlach W, Grama A, Harrison T, et al. MG-RAST version 4-lessons learned from a decade of low-budget ultra-high-throughput metagenome analysis. Brief Bioinform. 2019;20(4):1151–9. doi: 10.1093/bib/bbx105 ; PubMed Central PMCID: PMC6781595. 29028869 PMC6781595

[38] Zhao W, Wang Y, Liu S, Huang J, Zhai Z, He C, et al. The dynamic distribution of porcine microbiota across different ages and gastrointestinal tract segments. PLoS One. 2015;10(2):e0117441. Epub 2015/02/18. doi: 10.1371/journal.pone.0117441 ; PubMed Central PMCID: PMC4331431. 25688558 PMC4331431

[39] Gresse R, Chaucheyras Durand F, Duniere L, Blanquet-Diot S, Forano E. Microbiota composition and functional profiling throughout the gastrointestinal tract of commercial weaning piglets. Microorganisms. 2019;7(9). Epub 2019/09/25. doi: 10.3390/microorganisms7090343 ; PubMed Central PMCID: PMC6780805. 31547478 PMC6780805

[40] Li P, Niu Q, Wei Q, Zhang Y, Ma X, Kim SW, et al. Microbial shifts in the porcine distal gut in response to diets supplemented with *Enterococcus Faecalis* as alternatives to antibiotics. Sci Rep. 2017;7:41395. Epub 2017/02/07. doi: 10.1038/srep41395 ; PubMed Central PMCID: PMC5292720. 28165001 PMC5292720

[41] Chen X, Xu J, Ren E, Su Y, Zhu W. Co-occurrence of early gut colonization in neonatal piglets with microbiota in the maternal and surrounding delivery environments. Anaerobe. 2018;49:30–40. Epub 2017/12/11. doi: 10.1016/j.anaerobe.2017.12.002 . 29223548

[42] Kang E, Crouse A, Chevallier L, Pontier SM, Alzahrani A, Silue N, et al. Enterobacteria and host resistance to infection. Mamm Genome. 2018;29(7–8):558–76. Epub 2018/05/23. doi: 10.1007/s00335-018-9749-4 . 29785663

[43] Liu X, Mao B, Gu J, Wu J, Cui S, Wang G, et al. *Blautia*-a new functional genus with potential probiotic properties? Gut Microbes. 2021;13(1):1–21. Epub 2021/02/03. doi: 10.1080/19490976.2021.1875796 ; PubMed Central PMCID: PMC7872077. 33525961 PMC7872077

[44] Jiang X-l, Su Y, Zhu W-y. Fermentation characteristics of *Megasphaera elsdenii* J6 derived from pig feces on different lactate isomers. Journal of Integrative Agriculture. 2016;15(7):1575–83. doi: 10.1016/s2095-3119(15)61236-9

[45] Niu Q, Li P, Hao S, Zhang Y, Kim SW, Li H, et al. Dynamic distribution of the gut microbiota and the relationship with apparent crude fiber digestibility and growth stages in pigs. Sci Rep. 2015;5:9938. Epub 2015/04/22. doi: 10.1038/srep09938 ; PubMed Central PMCID: PMC4404679. 25898122 PMC4404679

[46] He X, Zhao S, Li Y, Chen T. *Faecalibacterium prausnitzii*: A next-generation probiotic in gut disease improvement. Canadian Journal of Infectious Diseases and Medical Microbiology. 2021;2021:1–10. doi: 10.1155/2021/6666114

[47] Suriyaphol P, Chiu JKH, Yimpring N, Tunsagool P, Mhuantong W, Chuanchuen R, et al. Dynamics of the fecal microbiome and antimicrobial resistome in commercial piglets during the weaning period. Sci Rep. 2021;11(1):18091. Epub 2021/09/12. doi: 10.1038/s41598-021-97586-9 ; PubMed Central PMCID: PMC8433359. 34508122 PMC8433359

[48] Zeineldin M, Aldridge B, Lowe J. Antimicrobial effects on swine gastrointestinal microbiota and their accompanying antibiotic resistome. Front Microbiol. 2019;10:1035. Epub 2019/06/04. doi: 10.3389/fmicb.2019.01035 ; PubMed Central PMCID: PMC6530630. 31156580 PMC6530630

[49] Lugsomya K, Yindee J, Niyomtham W, Tribuddharat C, Tummaruk P, Hampson DJ, et al. Antimicrobial resistance in commensal *Escherichia coli* isolated from pigs and pork derived from farms either routinely using or not using in-feed antimicrobials. Microb Drug Resist. 2018;24(7):1054–66. Epub 2018/09/12. doi: 10.1089/mdr.2018.0154 ; PubMed Central PMCID: PMC6154756. 30204569 PMC6154756

[50] WHO. Critically important antimicrobials for human medicine, 6th revision. Geneva, Switzerland: World Health Organization, 2019.

[51] Iannelli F, Santoro F, Santagati M, Docquier JD, Lazzeri E, Pastore G, et al. Type M resistance to macrolides is due to a two-gene efflux transport system of the ATP-binding cassette (ABC) superfamily. Front Microbiol. 2018;9:1670. Epub 2018/08/16. doi: 10.3389/fmicb.2018.01670 ; PubMed Central PMCID: PMC6079230. 30108557 PMC6079230

[52] Niestepski S, Harnisz M, Korzeniewska E, Aguilera-Arreola MG, Contreras-Rodriguez A, Filipkowska Z, et al. The emergence of antimicrobial resistance in environmental strains of the *Bacteroides fragilis* group. Environ Int. 2019;124:408–19. Epub 2019/01/27. doi: 10.1016/j.envint.2018.12.056 . 30682596

[53] Yu Z, Gunn L, Wall P, Fanning S. Antimicrobial resistance and its association with tolerance to heavy metals in agriculture production. Food Microbiol. 2017;64:23–32. Epub 2017/02/19. doi: 10.1016/j.fm.2016.12.009 . 28213031

[54] Zhang Y, Zhou J, Dong Z, Li G, Wang J, Li Y, et al. Effect of dietary copper on intestinal microbiota and antimicrobial resistance profiles of *Escherichia coli* in weaned piglets. Front Microbiol. 2019;10:2808. Epub 2020/01/11. doi: 10.3389/fmicb.2019.02808 ; PubMed Central PMCID: PMC6927916. 31921011 PMC6927916

[55] Cheng G, Ning J, Ahmed S, Huang J, Ullah R, An B, et al. Selection and dissemination of antimicrobial resistance in Agri-food production. Antimicrob Resist Infect Control. 2019;8:158. Epub 2019/10/28. doi: 10.1186/s13756-019-0623-2 ; PubMed Central PMCID: PMC6805589. 31649815 PMC6805589

[56] Paul D, Chakraborty R, Mandal SM. Biocides and health-care agents are more than just antibiotics: Inducing cross to co-resistance in microbes. Ecotoxicol Environ Saf. 2019;174:601–10. Epub 2019/03/16. doi: 10.1016/j.ecoenv.2019.02.083 . 30875553

[57] Wang C, Song Y, Tang N, Zhang G, Leclercq SO, Feng J. The shared resistome of human and pig microbiota is mobilized by distinct genetic elements. Appl Environ Microbiol. 2020. Epub 2020/12/15. doi: 10.1128/AEM.01910-20 ; PubMed Central PMCID: PMC8090867. 33310720 PMC8090867

[58] Rozwandowicz M, Brouwer MSM, Fischer J, Wagenaar JA, Gonzalez-Zorn B, Guerra B, et al. Plasmids carrying antimicrobial resistance genes in *Enterobacteriaceae*. J Antimicrob Chemother. 2018;73(5):1121–37. Epub 2018/01/26. doi: 10.1093/jac/dkx488 . 29370371

[59] Sun J, Fang LX, Wu Z, Deng H, Yang RS, Li XP, et al. Genetic analysis of the IncX4 plasmids: Implications for a unique pattern in the *mcr-*1 acquisition. Sci Rep. 2017;7(1):424. Epub 2017/03/25. doi: 10.1038/s41598-017-00095-x ; PubMed Central PMCID: PMC5428312. 28336940 PMC5428312

[60] Deng Y, Bao X, Ji L, Chen L, Liu J, Miao J, et al. Resistance integrons: class 1, 2 and 3 integrons. Ann Clin Microbiol Antimicrob. 2015;14:45. Epub 2015/10/22. doi: 10.1186/s12941-015-0100-6 ; PubMed Central PMCID: PMC4618277. 26487554 PMC4618277

[61] Pathirana H, Shin GW, Wimalasena S, Hossain S, De Silva BCJ, Dahanayake PS, et al. Incidence of antibiogram, antibiotic resistance genes and class 1 and 2 integrons in tribe *Proteeae* with IMP27 gene for the first time in *Providencia* sp. isolated from pet turtles. Lett Appl Microbiol. 2018;67(6):620–7. Epub 2018/09/22. doi: 10.1111/lam.13077 . 30238643

[62] Vandecraen J, Chandler M, Aertsen A, Van Houdt R. The impact of insertion sequences on bacterial genome plasticity and adaptability. Crit Rev Microbiol. 2017;43(6):709–30. Epub 2017/04/15. doi: 10.1080/1040841X.2017.1303661 . 28407717

[63] Zhu LH, Zhao KL, Chen XL, Xu JX. Impact of weaning and an antioxidant blend on intestinal barrier function and antioxidant status in pigs. J Anim Sci. 2012;90(8):2581–9. Epub 2012/08/17. doi: 10.2527/jas.2012-4444 . 22896732

[64] Wang J, Ji HF, Wang SX, Zhang DY, Liu H, Shan DC, et al. *Lactobacillus plantarum* ZLP001: *In vitro* assessment of antioxidant capacity and effect on growth performance and antioxidant status in weaning piglets. Asian-Australas J Anim Sci. 2012;25(8):1153–8. Epub 2012/08/01. doi: 10.5713/ajas.2012.12079 ; PubMed Central PMCID: PMC4093004. 25049675 PMC4093004

[65] Wang H, Xu R, Zhang H, Su Y, Zhu W. Swine gut microbiota and its interaction with host nutrient metabolism. Anim Nutr. 2020;6(4):410–20. Epub 2020/12/29. doi: 10.1016/j.aninu.2020.10.002 ; PubMed Central PMCID: PMC7750828. 33364457 PMC7750828

[66] Yang Z, Liao SF. Physiological effects of dietary amino acids on gut health and functions of swine. Front Vet Sci. 2019;6:169. Epub 2019/06/28. doi: 10.3389/fvets.2019.00169 ; PubMed Central PMCID: PMC6579841. 31245390 PMC6579841

