# Supplementary material for: Metagenomic analysis of the gut microbiota in piglets either challenged or not with enterotoxigenic Escherichia coli reveals beneficial effects of probiotics on microbiome composition, resistome, digestive function and oxidative stress responses
Source: PLoS One. 2022 Jun 24;17(6):e0269959. doi: 10.1371/journal.pone.0269959 (PMC9231746; doi:10.1371/journal.pone.0269959)

**S1 Fig. Schematic of experimental design and sample collection.** D indicates day after birth and hpc refers to hours post ETEC challenge.


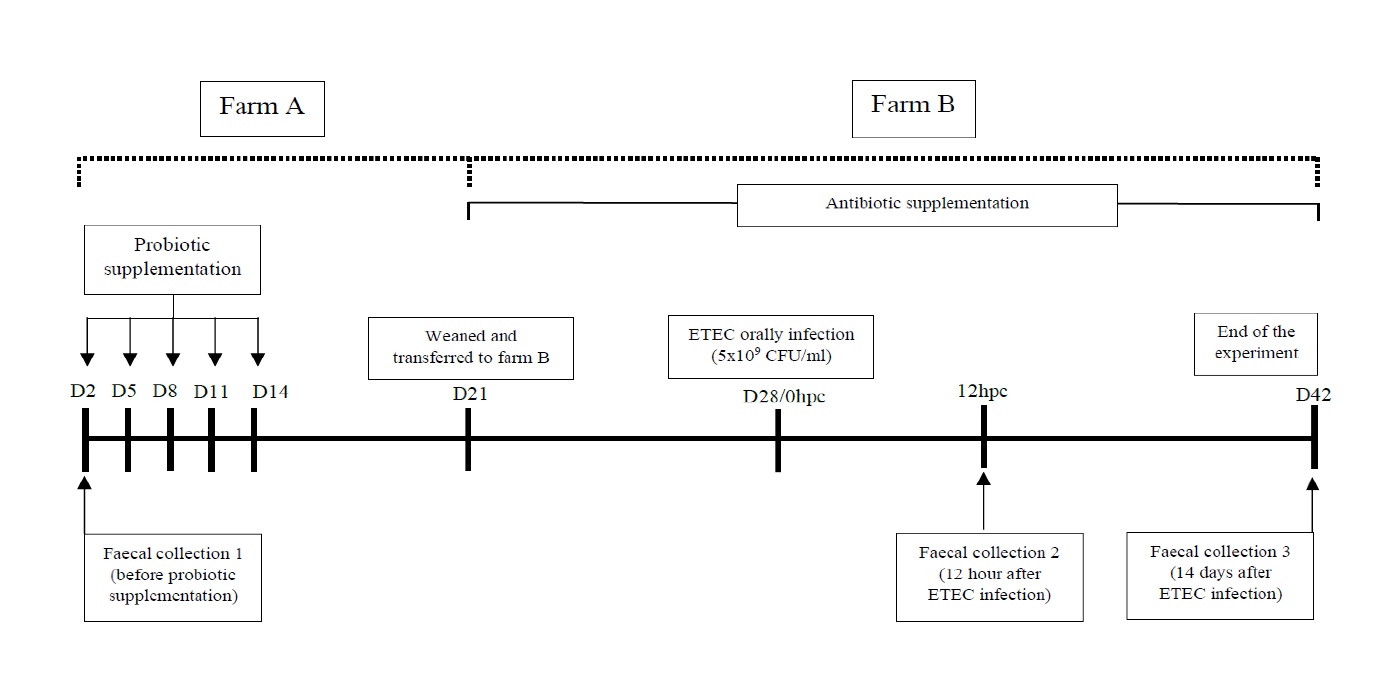

Supplement: S1 Fig — D indicates day after birth and hpc refers to hours post ETEC challenge. (DOCX) [file pone.0269959.s001.docx]
