# Supplementary material for: Metagenomic analysis of the gut microbiota in piglets either challenged or not with enterotoxigenic Escherichia coli reveals beneficial effects of probiotics on microbiome composition, resistome, digestive function and oxidative stress responses
Source: PLoS One. 2022 Jun 24;17(6):e0269959. doi: 10.1371/journal.pone.0269959 (PMC9231746; doi:10.1371/journal.pone.0269959)

**S2 Fig.** **Principal coordinate analysis (PCoA) plot based on Bray-Curtis dissimilarity index of microbial taxonomic profile at the species level from piglet faecal samples across treatments in each time-point.** The geometric shapes demonstrate the group of samples in each time-point. D2 refers to 2 days of age, before probiotic treatment.


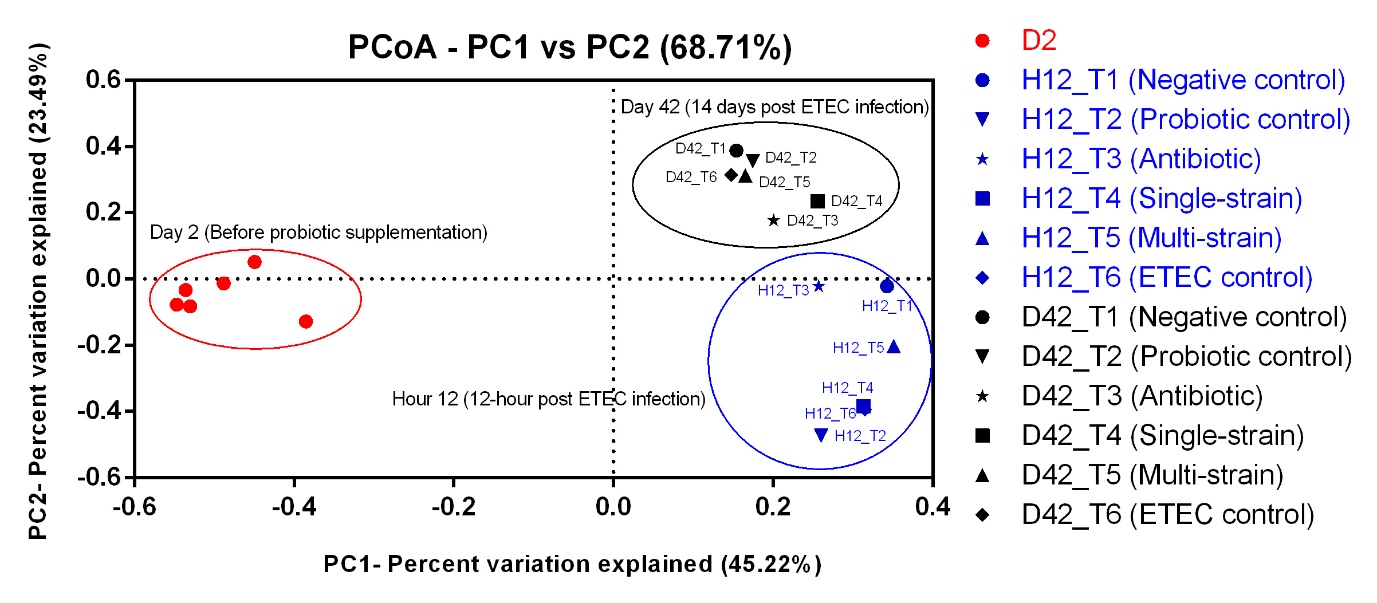

Supplement: S2 Fig — The geometric shapes demonstrate the group of samples in each time-point. D2 refers to 2 days of age, before probiotic treatment. (DOCX) [file pone.0269959.s002.docx]
