# Supplementary material for: Metagenomic analysis of the gut microbiota in piglets either challenged or not with enterotoxigenic Escherichia coli reveals beneficial effects of probiotics on microbiome composition, resistome, digestive function and oxidative stress responses
Source: PLoS One. 2022 Jun 24;17(6):e0269959. doi: 10.1371/journal.pone.0269959 (PMC9231746; doi:10.1371/journal.pone.0269959)

**S4 Fig. The relative abundance of the level 1 KEGG functional genes related to metabolism from piglet faecal samples in ETEC or non-ETEC infected piglets.** D2 refers to 2 days of age, before probiotic treatment.


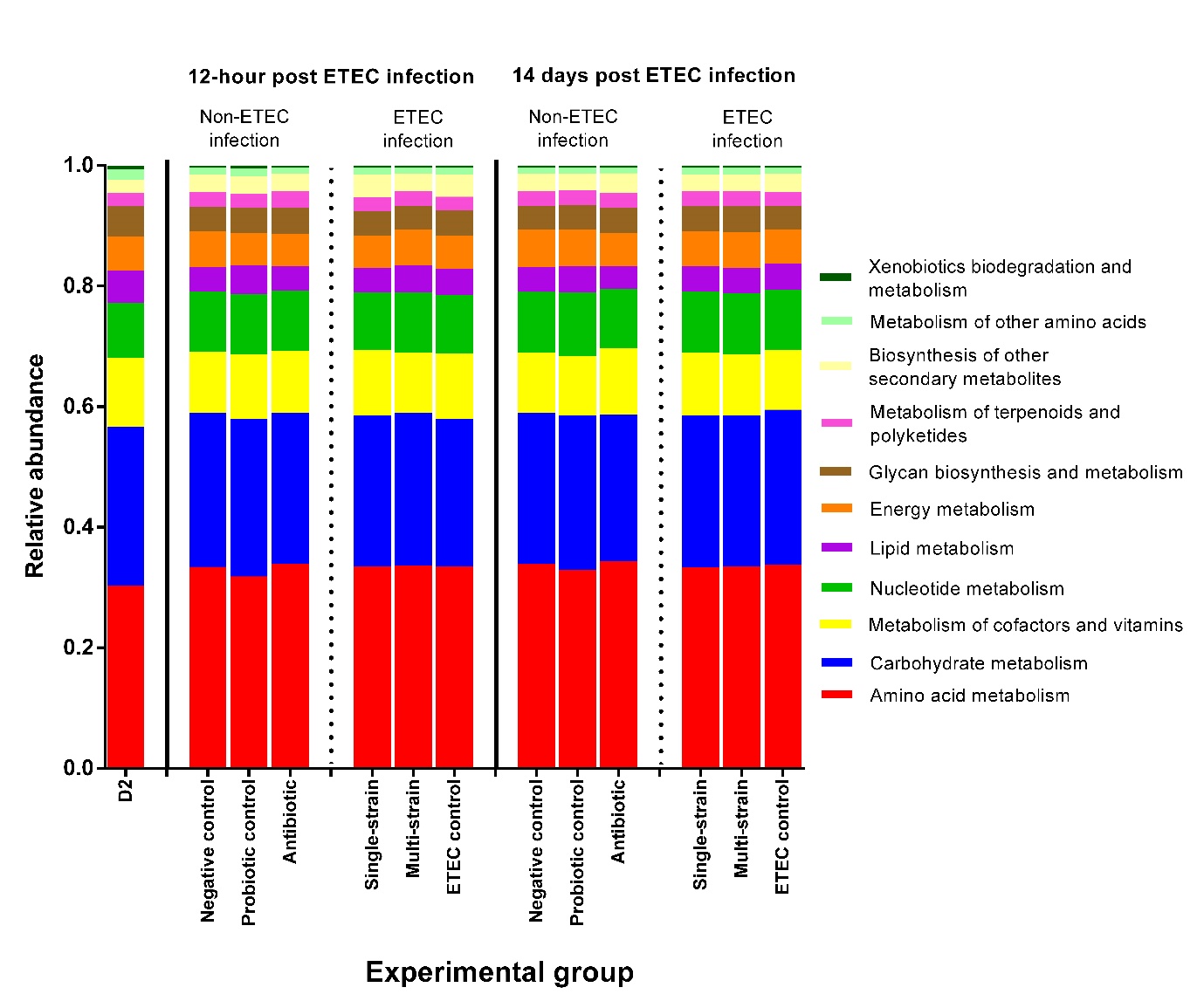

Supplement: S4 Fig — D2 refers to 2 days of age, before probiotic treatment. (DOCX) [file pone.0269959.s004.docx]
