# Supplementary material for: Metagenomic analysis of the gut microbiota in piglets either challenged or not with enterotoxigenic Escherichia coli reveals beneficial effects of probiotics on microbiome composition, resistome, digestive function and oxidative stress responses
Source: PLoS One. 2022 Jun 24;17(6):e0269959. doi: 10.1371/journal.pone.0269959 (PMC9231746; doi:10.1371/journal.pone.0269959)
Supplement: S1 Table — (DOCX) [file pone.0269959.s005.docx]

**S1 Table. Ingredient composition and nutrient concentration of the experimental basal diet.**

| **Ingredient Composition** | **Percent of dry matter** | |
| --- | --- | --- |
| Corn (7.7 % CP) | 26.75 | |
| Broken rice (7.7 % CP) | 22.97 | |
| Dehulled-soybean meal (48.9 % CP) | 18.47 | |
| Full fat soybean (36.0 % CP) | 10.00 | |
| Fish meal (60 % CP) | 6.00 | |
| Rice bran, full fat (13.6 % CP) | 5.00 | |
| Whey powder sweet | 5.00 | |
| Soybean oil | 2.67 | |
| Mono-Dicalcium Phosphate (MDCP; P 18.0 %, Ca 21.8 %) | 1.29 | |
| SP Premix (vitamin A 18,000 IU, vitamin D_3_ 2500 IU, vitamin E  250 IU, vitamin K_3_ 0.60 mg, vitamin B_1_ 3.2 mg, vitamin B_2_ 9.4 mg,  vitamin B_6_ 5 mg, vitamin B_12_ 80 µg, biotin 80 µg, choline 550 mg,  folic acid 2.15 mg, *D*-pantothenic acid 25 mg, nicotinic acid 75 mg, Ca 55 mg, Co 148 mg, Fe 148 mg, I-4.3 mg, Mn 80 mg and  Se 0.60 mg) | 0.50 | |
| Pellet binder | 0.30 | |
| L-Lysine HCl | 0.27 | |
| Sodium chloride | 0.23 | |
| Limestone (Ca 36.4%) | 0.20 | |
| DL-Methionine | 0.15 | |
| L-Threonine | 0.15 | |
| L-Tryptophan | 0.05 | |
| Total | 100.00 | |
| **Nutrient concentration** |  | **Unit** |
| Crude protein | 20.40 | % |
| Crude fat | 7.50 | % |
| Ashes | 6.89 | % |
| Crude fiber | 4.23 | % |
| Standardized ileal digestible Lysine:Metabolisable Energy (SID Lysine:ME) | 3.83 | g/Mcal |
| Digestible Lysine | 1.33 | % |
| Digestible Threonine | 0.83 | % |
| Calcium | 0.82 | % |
| Digestible Methionine + Cysteine | 0.76 | % |
| Phosphorus | 0.54 | % |
| Digestible Methionine | 0.49 | % |
| Digestible Tryptophan | 0.27 | % |
