# Supplementary material for: Metagenomic analysis of the gut microbiota in piglets either challenged or not with enterotoxigenic Escherichia coli reveals beneficial effects of probiotics on microbiome composition, resistome, digestive function and oxidative stress responses
Source: PLoS One. 2022 Jun 24;17(6):e0269959. doi: 10.1371/journal.pone.0269959 (PMC9231746; doi:10.1371/journal.pone.0269959)
Supplement: S2 Table — (DOCX) [file pone.0269959.s006.docx]

**S2 Table. Summary of overall sequencing data.**

| **Groups** | **Raw reads (bp)** | **Filter reads (bp)** | **Clean reads (%) ^ѱ^** |
| --- | --- | --- | --- |
| D2 | 74,120,140 | 62,794,856 | 84.72 |
|  | 72,871,648 | 60,319,200 | 82.77 |
|  | 73,524,896 | 59,669,194 | 81.16 |
|  | 74,116,640 | 65,053,102 | 87.77 |
|  | 72,232,588 | 60,155,428 | 83.28 |
|  | 78,739,346 | 64,092,260 | 81.40 |
| **12-hours post ETEC challenging** | | | |
| Non-ETEC infection | | | |
| Negative control | 71,742,904 | 67,131,364 | 93.57 |
| Probiotic control | 68,918,602 | 64,408,060 | 93.46 |
| Antibiotic | 71,224,186 | 66,434,034 | 93.27 |
| ETEC infection | | | |
| Single-strain | 72,708,420 | 68,426,454 | 94.11 |
| Multi-strain | 71,052,434 | 66,982,406 | 94.27 |
| ETEC control | 88,132,182 | 82,394,492 | 93.49 |
| **14-days post ETEC challenging** | | | |
| Non-ETEC infection | | | |
| Negative control | 76,764,072 | 69,651,048 | 90.73 |
| Probiotic control | 115,242,034 | 109,361,858 | 94.90 |
| Antibiotic | 77,712,366 | 67,600,042 | 86.99 |
| ETEC infection | | | |
| Single-strain | 77,700,814 | 74,171,530 | 95.46 |
| Multi-strain | 82,498,506 | 74,089,966 | 89.81 |
| ETEC control | 86,952,230 | 77,731,060 | 89.40 |

**^ѱ^** Clean reads were calculated as (Filter reads/Raw reads) x 100. D2 refers to 2 days of age, before probiotic treatment.
