# Supplementary material for: Metagenomic analysis of the gut microbiota in piglets either challenged or not with enterotoxigenic Escherichia coli reveals beneficial effects of probiotics on microbiome composition, resistome, digestive function and oxidative stress responses
Source: PLoS One. 2022 Jun 24;17(6):e0269959. doi: 10.1371/journal.pone.0269959 (PMC9231746; doi:10.1371/journal.pone.0269959)
Supplement: S3 Table — (DOCX) [file pone.0269959.s007.docx]

**S3 Table. The summary of all *de novo* assembled metagenomic sequence data by using MEGAHIT and determining by QUAST.**

| **Groups** | **Assembled contigs** | **Total sequence length (bp)** | **N50** |
| --- | --- | --- | --- |
| D2 | 347,741 | 196,215,439 | 749 |
|  | 228,884 | 154,065,378 | 840 |
|  | 257,749 | 166,668,686 | 821 |
|  | 242,934 | 166,548,412 | 848 |
|  | 219,197 | 155,302,907 | 1,169 |
|  | 259,329 | 166,280,013 | 830 |
| **12-hours post ETEC challenging** | | | |
| Non-ETEC infection | | | |
| Negative control | 355,198 | 448,048,080 | 3,885 |
| Probiotic control | 133,927 | 153,868,780 | 3,707 |
| Antibiotic | 624,196 | 668,618,351 | 2,419 |
| ETEC infection | | | |
| Single-strain | 207,869 | 268,616,577 | 3,588 |
| Multi-strain | 280,248 | 347,364,359 | 3,762 |
| ETEC control | 214,206 | 283,634,843 | 3,889 |
| **14-days post ETEC challenging** | | | |
| Non-ETEC infection | | | |
| Negative control | 323,166 | 411,426,419 | 3,779 |
| Probiotic control | 323,707 | 504,325,326 | 7,425 |
| Antibiotic | 358,033 | 300,817,974 | 2,618 |
| ETEC infection | | | |
| Single-strain | 430,156 | 528,248,353 | 3,403 |
| Multi-strain | 443,347 | 566,818,604 | 3,686 |
| ETEC control | 318,388 | 416,422,550 | 4,425 |

D2 refers to 2 days of age, before probiotic treatment.
