# Supplementary material for: Metagenomic analysis of the gut microbiota in piglets either challenged or not with enterotoxigenic Escherichia coli reveals beneficial effects of probiotics on microbiome composition, resistome, digestive function and oxidative stress responses
Source: PLoS One. 2022 Jun 24;17(6):e0269959. doi: 10.1371/journal.pone.0269959 (PMC9231746; doi:10.1371/journal.pone.0269959)
Supplement: S4 Table — (DOCX) [file pone.0269959.s008.docx]

**S4 Table. Alpha diversity of gut microbial communities from piglet faecal samples across treatments and each time-point.**

| **Groups** | **Species richness** | **Shannon** | **Simpson** |
| --- | --- | --- | --- |
| D2 | 5473 | 4.567 | 0.838 |
| **12-hours post ETEC challenging** | | | |
| Non-ETEC infection | | | |
| Negative control | 5410 | 5.218 | 0.875 |
| Probiotic control | 4882 | 2.471 | 0.549 |
| Antibiotic | 5433 | 6.259 | 0.914 |
| ETEC infection | | | |
| Single-strain | 5193 | 2.404 | 0.482 |
| Multi-strain | 5345 | 3.734 | 0.758 |
| ETEC control | 5243 | 2.430 | 0.481 |
| **14-days post ETEC challenging** | | | |
| Non-ETEC infection | | | |
| Negative control | 5511 | 7.894 | 0.969 |
| Probiotic control | 5489 | 7.364 | 0.956 |
| Antibiotic | 5500 | 7.956 | 0.977 |
| ETEC infection | | | |
| Single-strain | 5505 | 6.837 | 0.954 |
| Multi-strain | 5529 | 8.123 | 0.982 |
| ETEC control | 5499 | 7.295 | 0.972 |

D2 refers to 2 days of age, before probiotic treatment.
