# Supplementary material for: Metagenomic analysis of the gut microbiota in piglets either challenged or not with enterotoxigenic Escherichia coli reveals beneficial effects of probiotics on microbiome composition, resistome, digestive function and oxidative stress responses
Source: PLoS One. 2022 Jun 24;17(6):e0269959. doi: 10.1371/journal.pone.0269959 (PMC9231746; doi:10.1371/journal.pone.0269959)
Supplement: Attachment — Submitted filename: Response to the journal requirements.docx [file pone.0269959.s009.docx]

Dear Editor of PLOS ONE

Thank you very much for your suggestions to improve our manuscript " Metagenomic analysis of the gut microbiota in piglets either challenged or not with enterotoxigenic *Escherichia coli* reveals beneficial effects of probiotics on microbiome composition, resistome, digestive function and oxidative stress responses ". We appreciate your kind response and the reviewer’s extensive analysis. All suggestions have been carefully responded to topic by topic and noted in yellow outlining and blue lettering in the revised manuscript. We hope that our revisions will be satisfactory to merit publication in PLOS ONE.

Sincerely yours,

Prapasarakul N.

Corresponding author

**Journal Requirements**

**When submitting your revision, we need you to address these additional requirements.**

1. Please ensure that your manuscript meets PLOS ONE's style requirements, including those for file naming

We have checked throughout the manuscript and believed that this revised manuscript now meets the PLOS ONE style requirements.

2. In your Methods section, please include a comment about the state of the animals following this research. Were they euthanized or housed for use in further research? If any animals were sacrificed by the authors, please include the method of euthanasia, and describe any efforts that were undertaken to reduce animal suffering.

All the experimental piglets were sacrificed at the end of the experiment (at 42 days of age). We have provided the required information under “Animals and housing” (Page 5-6, Materials and methods section, Lines 117-121).

3. Thank you for stating the following in the Acknowledgments Section of your manuscript:

“This research was also supported by Pathogen Bank Project, Chulalongkorn Academic Advancement into Its 2nd Century Project and the CHE-TRF Senior Research Fund (RTA6280013).”

We note that you have provided additional information within the Acknowledgements Section that is not currently declared in your Funding Statement. Please note that funding information should not appear in the Acknowledgments section or other areas of your manuscript. We will only publish funding information present in the Funding Statement section of the online submission form.

Please remove any funding-related text from the manuscript and let us know how you would like to update your Funding Statement. Currently, your Funding Statement reads as follows:

“The funders had no role in study design, data collection and analysis, decision to publish, or preparation of the manuscript.”

Please include your amended statements within your cover letter; we will change the online submission form on your behalf.

Thank you for your comment. In the revised version, we have removed any funding-related text according to your recommendation. Moreover, funding information have entered in the financial disclosure section of the submission system.

4. Please include captions for your Supporting Information files at the end of your manuscript, and update any in-text citations to match accordingly

Thank you for your comment. We have revised about captions in the supporting information according to your recommendation.
